# Molecular Linkage between Immune System Disorders and Atherosclerosis

**DOI:** 10.3390/cimb45110552

**Published:** 2023-11-01

**Authors:** Katarzyna Napiórkowska-Baran, Oskar Schmidt, Bartłomiej Szymczak, Jakub Lubański, Agata Doligalska, Zbigniew Bartuzi

**Affiliations:** 1 Department of Allergology, Clinical Immunology and Internal Diseases, Collegium Medicum Bydgoszcz, Nicolaus Copernicus University Toruń, 85-067 Bydgoszcz, Poland; zbartuzi@cm.umk.pl; 2 Student Research Club of Clinical Immunology, Department of Allergology, Clinical Immunology and Internal Diseases, Collegium Medicum Bydgoszcz, Nicolaus Copernicus University Toruń, 85-067 Bydgoszcz, Poland; okischmidt@wp.pl (O.S.); bartlomiej.szymczak1@gmail.com (B.S.); kuba.albanski@gmail.com (J.L.); doli285@wp.pl (A.D.)

**Keywords:** atherosclerosis, cardiovascular risk, immune system, inborn errors of immunity, primary immunodeficiencies, secondary immunodeficiencies, chronic kidney disease, autoimmunity, cancer, malnutrition

## Abstract

A strong relationship exists between immune dysfunction and cardiovascular disease. Immune dysregulation can promote the development of cardiovascular diseases as well as exacerbate their course. The disorders may occur due to the presence of primary immune defects (currently known as inborn errors of immunity) and the more common secondary immune deficiencies. Secondary immune deficiencies can be caused by certain chronic conditions (such as diabetes, chronic kidney disease, obesity, autoimmune diseases, or cancer), nutritional deficiencies (including both lack of nutrients and bioactive non-nutrient compounds), and medical treatments and addictive substances. This article unravels the molecular linkage between the aforementioned immune system disorders and atherosclerosis.

## 1. Introduction

Atherosclerosis is a disease that develops over years and mainly affects large and medium-sized arterial vessels. In its effect, vascular flow may be restricted as a result of long-term lipid accumulation in the walls of the vessels, thereby leading to a gradual, and occasionally sudden and life-threatening, reduction in the supply of oxygen and nutrients to the various organs, including the most important ones—namely, the heart and brain. Coronary heart disease (CHD), the cause of which is specifically atherosclerosis, is the leading cause of mortality in adults worldwide. The atherosclerotic process commences even before birth and progresses with age. Initially it develops without any symptoms. By the time the first symptoms appear, changes are already advanced. They can worsen gradually or occur suddenly, without prior warning. In recent years, substantial changes have been observed in the epidemiology of atherosclerosis. In the past, atherosclerosis occurred predominantly in older men; however, nowadays, its incidence has increased in women and younger people [1,2].

The role of the immune system is to maintain the organism’s homeostasis, especially in terms of protection against pathogens and other external environmental factors, but also to control and protect against the development of various diseases such as cancer, autoimmunity, and cardiovascular disease. There is a strong relationship between immune dysregulation and cardiovascular disease (CVD). Immune dysregulation can promote the development of CVDs, as well as exacerbate their course. The disorders might result from the presence of primary immune defects (currently known as inborn errors of immunity) and the more common secondary immune deficiencies. Secondary immune deficiencies can be caused by certain chronic conditions, such as diabetes, chronic kidney disease, obesity, autoimmune diseases, or cancer), nutritional deficiencies (including both nutrients and bioactive non-nutrient compounds), and medical treatments and addictive substances.

The aim of the review is to present the molecular linkage between immune system disorders and atherosclerosis, with particular emphasis on primary and secondary immunodeficiencies.

## 2. Etiopathogenesis of Atherosclerosis with Special Emphasis on the Role of the Immune System

Atherosclerosis is one of the most common cardiovascular diseases. It is a major cause of ischemic heart disease, cerebrovascular disease, peripheral vascular disease, and aneurysms. Traditionally, atherosclerosis was thought to be a disease associated with cholesterol accumulation caused by the retention of lipoproteins, including low-density lipoproteins (LDLs), in the lining of the arteries. We now know that atherosclerosis is a chronic inflammatory disease of the arteries, and the role of both genetic and environmental factors in its pathogenesis is emphasized. The etiopathogenesis of atherosclerosis is multifactorial and inextricably linked to the processes occurring within the immune system [3,4].

Atherosclerosis begins with damage to the endothelial cells of arteries as a result of exposure to factors such as hypertension, diabetes, and substances in cigarettes, which then leads to the penetration and slow accumulation of lipoproteins, particularly oxidized LDL cholesterol particles (oxLDL), into the wall of these vessels. At the site of injury, endothelial cells express adhesion molecules (mainly platelet endothelial cell adhesion molecule (PECAM), intercellular adhesion molecule-1 (ICAM-1), vascular cell adhesion molecule-1 (VCAM-1), and selectin) through which monocyte recruitment occurs. These cells, which migrate through the vessel wall, transform into two subtypes of macrophages depending on the microenvironment:

- Proinflammatory macrophages, as a result of exposure to oxidized lipid molecules, free fatty acids, and interferon gamma (IFN-γ). This type of macrophage is predominant in atherosclerotic plaque, and these cells, in addition to producing proinflammatory cytokines (interleukin-1 beta (IL-1β), interleukin-6 (IL-6)), engulf lipids, thus becoming foam cells, which, together with vascular smooth muscle cells, are responsible for the formation of a necrotic core inside the atherosclerotic plaque.
- Anti-inflammatory macrophages, as a result of exposure to interleukins 4, 10, and 13. These cells stimulate collagen production and efficient removal of lipid particles from the arterial wall, and are also involved in other processes associated with regression of atherosclerosis [4,5].

In addition to monocytes, T-lymphocytes also play an important role in the development of atherosclerosis, as they are activated by antigens such as oxLDL, apolipoprotein B (ApoB), and heat shock protein (HSP) presented by antigen-presenting cells (APCs). Initially, naive T cells migrate from lymphoid tissues to the atherosclerotic plaque, where they undergo selective activation and, in the vast majority, clonal expansion toward atherogenic type 1 helper (Th1), and a smaller proportion toward atheroprotective types such as regulatory T cell (Treg) and other types such as Th2 and Th17, the presence of which has been identified in atherosclerotic plaques [4,5,6,7].

As previously mentioned, the predominant cells in atherosclerotic plaques are Th1 cells, which are directly associated with atherogenesis due to their production of IFN-γ (increases recruitment of macrophages and T cells, promotes foam cell formation and production of proinflammatory cytokines, decreases plaque stability) and tumor necrosis factor alpha (TNF-α; recruitment of leukocytes, production of proinflammatory cytokines and enhancement of oxidative stress). In contrast, Treg cells, which have an atheroprotective function, release transforming growth factor beta (TGF-β increases plaque stability, inhibits recruitment and activation of T cells and macrophages) and IL-10 (reduces T cell differentiation and cytokine secretion from T cells and macrophages). The role of other types of T lymphocytes in the development of atherosclerosis remains poorly understood and is currently under investigation [4,5,6,7].

**Figure 1:**
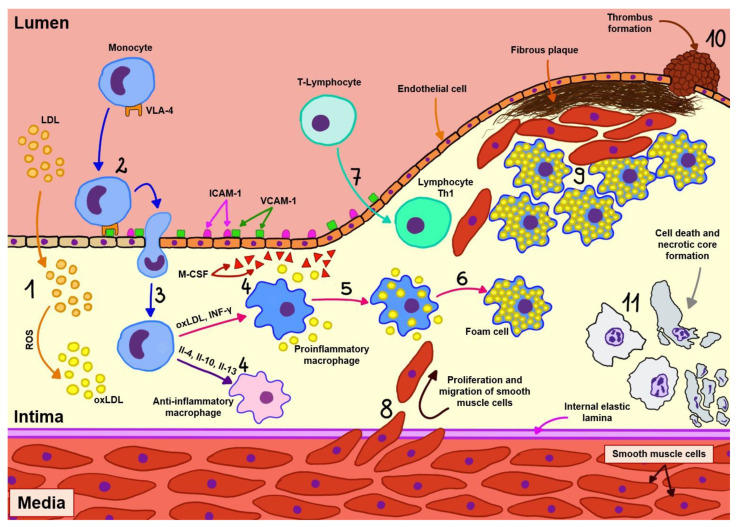
Pathophysiological mechanism of atherosclerosis. (1) LDL particles penetrate deep into the arterial wall, where they are oxidized by reactive oxygen species (ROS) to oxLDL. (2) At the site of arterial endothelial injury, monocyte recruitment occurs. (3) The VLA-4 receptor binds to the adhesion molecules, allowing monocytes to migrate into the arterial wall. (4) Depending on the microenvironment, monocytes transform into either proinflammatory or anti-inflammatory macrophages. (5) Proinflammatory macrophages engulf oxLDL particles, while the locally produced macrophage colony-stimulating factor (M-CSF) enhances their chemotactic and phagocytic activity. (6) Macrophages packed with oxLDL become foam cells. (7) Naive T-lymphocytes migrate into the atherosclerotic plaque, where their activation and clonal expansion mainly into Th1 lymphocytes ensues. (8) Vascular smooth muscle cells proliferate and migrate to the forming core of the atherosclerotic plaque. (9) Over time, atherosclerotic plaque builds up in the vessel wall. (10) Thrombus forms at the site of plaque rupture. (11) Ultimately, the cells disintegrate, forming a necrotic core.

The aforementioned cells, with the accumulated cholesterol, form an enlarging atherosclerotic plaque in the vessel wall. As it develops, blood flow through the vessel decreases and the connective tissue degrades, weakening the structure of the plaque. The positive vascular remodeling occurring in more active lesions destabilizes atherosclerotic plaques. This may lead to rupture of the plaque and the formation of a thrombus, which causes closure of the artery and sudden ischemia of the area supplied by it. In the end, such a condition manifests itself as acute coronary syndrome or stroke. In addition, in advanced fibrotic lesions, negative vascular remodeling transpires. The contents of the plaque bulge inside the vessel, exacerbating the stenosis and leading to chronic coronary syndromes [4,6,8]. The pathogenesis of atherosclerosis is presented in Figure 1.

Although epigenetic factors are salient in the pathogenesis of atherosclerosis, genetic factors should not be forgotten. Genetic determinants of atherosclerosis have a polygenic character. The most significant genes related to lipid metabolism are the apolipoprotein B (ApoB) and apolipoprotein E (ApoE) genes, the lipoprotein lipase (LPL) genes, and the cholesterol ester transfer protein (CETP) gene. Individuals who have inherited the APOE2 allele from both parents carry an increased risk of atherosclerotic disease and elevated cholesterol levels. Having at least one copy of the APOE4 allele, on the other hand, is also associated with an increased risk of atherosclerosis [9]. Familial hypercholesterolemia is usually caused by mutations in the gene encoding the low-density lipoprotein receptor (LDLR) and genes encoding receptor-interacting proteins: APOB, PCSK9, and LDLRAP1. Monogenic hypertriglyceridemia is the result of mutations in the genes regulating the metabolism of triglyceride-rich lipoproteins [10]. The development of atherosclerosis is also favored by mutations of genes regulating platelet function, coagulation, and fibrinolysis, including genes encoding glycoprotein GPIIIa, GPIIb, or the glycoprotein IIa/IIIb complex [11]. Not less important are also alterations of the DNA sequences regulating the inflammatory process—genes for interleukins (especially IL-6), P and E selectins, CD14, and adhesion molecules [12]. Recent studies indicate that autophagy-related genes (ARGs) also play a role in the pathogenesis of atherosclerosis [13].

## 3. Inborn Errors of Immunity and Atherosclerosis

Inborn errors of immunity (IEI), formerly known as primary immunodeficiencies (PIDs), are a group of disorders in which there is an increased susceptibility to infection, autoimmune disease, and cancer. It is estimated that 70–80% of patients with PID remain undiagnosed and are not included in patient registries. The delay in diagnosis is up to 10 years, resulting in the occurrence of numerous complications, including death [14]. Therefore, current trends in clinical immunology focus on establishing an accurate diagnosis as soon as possible, which determines the length and quality of life. According to the Expert Committee of the International Union of Immunological Societies, the number of IEI disease entities has increased from 150 in 2007 to more than 485 today [15]. Along with the improved diagnosis of patients with IEI, their cardiovascular risk is increasing, but unfortunately this issue is still underestimated.

In patients with IEI, the impact on the progression of atherosclerosis is multifactorial. It results not only from the genetic defect, but also from the presence of concomitant conditions, such as infection, autoimmunity, or cancer. Immune dysregulation may be further exacerbated by the treatment used. The primary immune defect may involve cellular immunity, humoral immunity, phagocytosis, the complement system, bone marrow failure, or may be the result of the occurrence of any of these in isolation or in combination.

The available literature focuses mainly on patients with common variable immunodeficiency (CVID), which belongs to deficiencies associated with antibody defects. A study by Andrade IGA et al. showed a greater incidence of high levels of ultrasensitive C-reactive protein (CRP), reduced amount selenium in plasma and glutathione peroxidase (GPX) activity, elevated oxidized LDL fraction, and decreased levels of Apo-A-1 in CVID patients compared to the control group, suggesting oxidative stress and increased cardiovascular risk [16]. Chronic reduction in circulating immunoglobulin G (IgG) is associated with impairment of endothelium-mediated vascular reactivity. The available literature has shown that IgG directly stimulates endothelial cells and increases nitrogen oxide (NO) production, which has been reported in vivo, as well as in isolated arterial endothelial cells in vitro [17]. The author’s study of 94 adult patients with primary antibody deficiency showed that as many as 72.5% had impaired lipid metabolism, and the mean number of cardiovascular risk factors was 5 ± 3 (unpublished data).

Chronic granulomatous disease, or CGD in short, is a hereditary immunodeficiency caused by mutations in genes that encode the key components of the phagocyte NADPH oxidase (known as NOX2), gp91phox, p22phox, p47phox, p67phox, and seldom p40phox, resulting in impaired formation of superoxide anion and other reactive oxygen intermediates. Reduced levels of residual reactive oxygen species (ROS) synthesis by neutrophils are associated with premature mortality. Considerably greater formation of proinflammatory mediators by CGD myeloid cells (e.g., IL-8, leukotriene B4 (LTB4)), and reduced apoptosis of neutrophils are also believed to be prominent components of inordinate secondary inflammation [18]. Inflammatory cells such as neutrophils chronically damage tissues in inflammation. In order to curb this process, programmed cell death, or apoptosis, is necessary. When this process occurs at a lower frequency, accumulated effector cells such as neutrophils may contribute to prolonging inflammatory reactions [19].

**Figure 2:**
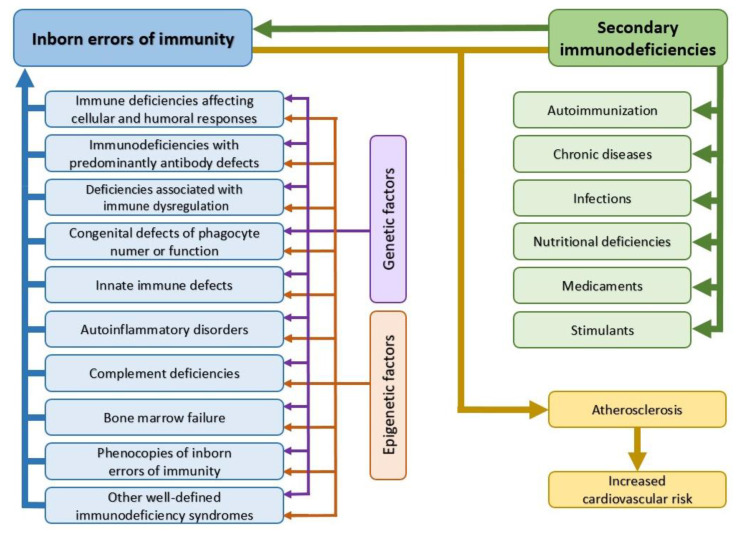
The relationship between inborn errors of immunity, secondary immunodeficiencies and atherosclerosis.

Given the rapid advances in knowledge about IEI, it is only a matter of time before individual immune deficiencies will be thoroughly analyzed in relation to the pathogenesis of atherosclerosis. The relationship between IEIs, secondary immunodeficiencies, and atherosclerosis is shown in Figure 2.

## 4. The Role of Secondary Immunodeficiencies in the Atherosclerosis Development

Secondary immunodeficiencies are much more common than primary immunodeficiencies. Their incidence is greatly underestimated and is often associated with specific disease entities. They can be the result of chronic conditions, such as diabetes, chronic kidney disease, autoimmunity, or cancer, as well as the result of malnutrition, medical treatment, or addictive substances.

### 4.1. Chronic Conditions

#### 4.1.1. Diabetes Mellitus

Insulin resistance (IR) is the most common reason for diagnosis of type 2 diabetes. The organism stimulates the pancreas to overproduce insulin in an effort to lower blood glucose levels. Overproduction of insulin leads to a phenomenon called hyperinsulinemia, which amplifies the deposition of atherosclerotic plaques by recruiting immune cells into the endothelium of blood vessels, stimulating inflammation of these vessels and the proliferation of vascular smooth muscle cells (VSMCs). Increased insulin production leads to the formation of excessive amounts of C-peptide, which arises from proinsulin during formation of its active form. It turns out that C-peptide also promotes VSMC proliferation. It uses its effect on the protein tyrosine kinase Src, the activation of which indirectly leads to the activation of the phosphoinositide 3-kinase (PI3K)/protein kinase B (AKT)/serine/threonine protein kinase (mTOR) and Ras/mitogen-activated protein kinase (MAPK) pathways, which can stimulate VSMC proliferation [20]. Serine/threonine protein kinase (mTOR) is found in protein complexes (mTORC1 and mTORC2). It is an instrumental regulator of cell growth and metabolism; hence, its activation in the PI3K/AKT pathway ultimately leads to VSMC proliferation [21,22,23]. Coletta et al. performed a 4-h insulin intervention on healthy volunteers and found increased inflammatory gene expression in muscle biopsies [24].

IR contributes to lipid metabolism disorders. It promotes excessive lipogenesis and increases the production of very-low-density lipoproteins (VLDL) [20]. In diabetes, low-density lipoproteins (LDL) are more easily oxidized, making them more susceptible to be uptaken by the endothelial cells, whereas components of the lipid panel with beneficial effects, such as high-density lipoprotein (HDL) and apolipoprotein A1, have reduced values [25].

Hyperglycemia induces protein glycation and glycoxidation. This results in the formation of advanced glycation end-products (AGEs), which activate the endothelium, increase the surface expression of adhesion molecules, and, consequently, interfere with signal transduction and the formation of reactive oxygen species (ROS). In addition to ROS, additional factors accelerating the formation of atherosclerotic plaque in diabetic patients include the observed increased levels of IL-1β, IL-18, increased inflammasomes activity, and neutrophil extracellular traps (NET) [25,26,27].

Using mouse models, Nagareddy et al. [26] showed that in hyperglycemic states, increased levels of circulating inflammatory Ly6C^+^ CCR2^+^ monocytes and neutrophils are observed. Diabetes stimulates the proliferation of granulocyte progenitor cells, macrophages, and myeloid cells in the bone marrow, without altering processes involving stem cells. In the mentioned study, elevated levels of granulocyte colony-stimulating factor (G-CSF) were found. However, due to reports indicating impaired effects of G-CSF in hyperglycemia, the authors also examined other factors that may cause changes in the number of the aforementioned leukocytes. The expression of DAMPs (damage-associated molecular patterns), which are associated with inflammatory processes, was increased in plasma, specifically S100A8, S100A9, and high mobility group box protein 1 (HMGB1). The expression of the former two decreased after lowering glucose levels. The DAMPs are responsible for the proliferation of bone marrow progenitor cells, while they are dependent on the receptor for advanced glycation end-products (RAGE) [25,26,27].

In the environment of elevated glucose levels, production of diacylglycerol (DAG) increases, which activates protein kinase C (PKC). PKC decreases NO production in endothelial cells by inhibiting endothelial NO synthase (eNOS), and increases vascular permeability and remodeling of the extracellular matrix [25,27]. Subsequent changes in the extracellular matrix also lead to increased interactions with RAGE on macrophages, endothelial cells, and VSMCs—resulting in persistent inflammation and the generation of reactive oxygen species. Hyperglycemia is a factor that increases organ perfusion. This leads to increased cellular metabolism and, consequently, to the formation of reactive oxygen species (ROS) in the mitochondria [27]. In diabetes, atherosclerotic plaques are more prone to ulceration. This effect is caused by matrix metalloproteinases 1 and 2 (MMP-1 and MMP-2), which are overactive in diabetes. This leads to sensitization of the atherosclerotic plaque and ultimately increases the risk of rupture and subsequent embolism [25].

Increased platelet activity is another dysfunction seen in patients with diabetes. There is an increase in the expression of thromboxane (TxA2) and von Willebrand factor. The treatment of the abovementioned disorders in these patients is further complicated by altered binding affinity of all receptors for antiplatelet drugs [25].

Osawa et al. used autofluorescence to measure skin AGE concentrations in type 1 diabetics and compared them with age- and sex-matched controls. Diabetic patients were proven to have a higher risk of atherosclerosis in the carotid arteries [28].

#### 4.1.2. Chronic Kidney Disease

The final stage of chronic kidney disease, end-stage renal disease (ESRD), is characterized by significantly higher cardiovascular mortality than in the general population. Severely impaired renal function, along with hemodialysis, has a significant impact on the development of atherosclerosis and heart disease such as congestive heart failure and left ventricular hypertrophy. Additionally, ESRD, or, colloquially, uremia, is a proinflammatory condition in which inflammation maintains contact between dialysis membranes and immune cells in the blood. As a result, proinflammatory cytokines are secreted in response to the recognition of foreign material. Another factor influencing activation of the immune system is the accumulation of toxic metabolites in anticipation of hemodialysis. The very type of dialysis performed (hemodialysis or hemofiltration and hemodiafiltration, due to the removal of products of different sizes: small and medium, respectively) also affects the activity of the immune system [29].

Platelet-derived growth factor (PDGF) is secreted by monocytes and, according to recent findings, is responsible for the initiation and maintenance of vascular inflammation. Another factor secreted by monocytes is vascular endothelial growth factor (VEGF), which is involved in the synthesis of nitric oxide in endothelial cells and is associated with hypervolemia, in which case it is produced in greater quantity. It enhances the formation and subsequent destabilization of atherosclerotic plaque. IL-6, known as a systemic indicator of inflammation, also plays a major role in maintaining the inflammatory state, which acts as a stimulus for the formation of atherosclerosis. Li et al. [30] observed that in the general elderly population, elevated levels of IL-6 correlate with death from cardiovascular causes. This also applies to hemodialysis patients, in whom cardiovascular pathologies account for a large percentage of deaths [30,31].

Monoamine oxidase A (MAO-A) and clusterin (apolipoprotein J) are also involved in the pathogenesis of atherosclerotic plaque through their involvement in the generation of oxidative stress [29].

Simone et al. compared patients treated with hemodialysis (HD) and hemodiafiltration (ol-HDF, on-line hemodiafiltration) in terms of the aforementioned factors leading to atherosclerotic lesions. Patients treated with ol-HDF showed lower expression of genes encoding PDGF, VEGF, IL-6, MAO-A, and clusterin. The authors also took into account the paracrine secretion of the factors, and therefore determined the serum concentrations of PDGF, IL-6, and VEGF in the same patients, where they also demonstrated lower values. In addition, in patients treated with HD, the dialysis regimen was changed to ol-HDF for 6 months, with the effect of lowering the parameters studied. The only difference was in the levels of apolipoprotein E (ApoE), which prevents the development of atherosclerosis. Its values were higher both in patients initially treated with ol-HDF and in those whose dialysis was switched to ol-HDF [29].

Patients with chronic kidney disease are characterized by a wide range of abnormalities in vascular endothelial function. One of the common abnormalities is an impaired vasodilation, which correlates with the severity of kidney disease. This is due to the reduced expression and activation of NO synthase, which is negatively affected by uremic toxins. The reduced ability to dilate the blood vessel results in increased pulse wave velocity in patients with chronic kidney disease. Increased velocity and turbulent blood flow adversely affect endothelial cell continuity. Parameters that reflect the state of the endothelium are increased amounts of circulating endothelial microparticles (EMPs), circulating endothelial cells (CECs) and decreased number of endothelial progenitor cells (EPCs). Uremic toxins induce activation of the nuclear factor kappa B (NF-κB) pathway and, consequently, structural disruption of cytoskeletal fibers and extracellular matrix. The effect of this process is a reduction in endothelial cell interaction. On detached endothelial cells, increased levels of endothelial cell adhesion molecules and von Willebrand factor are observed, which is the first inducer of thrombus formation, with atherosclerotic plaque on its basis [32].

Some reports indicate a positive effect of vitamin D3, which is known to be deficient in chronic kidney disease. The active form of the vitamin prevents endothelial cell detachment [32]. A reduction in the impairment of flow-mediated dilation (FMD) has been observed in patients with stages 3 and 4 of chronic kidney disease [33]. In contrast, hyperphosphatemia has a negative effect on the endothelium. Phosphorus stimulates atherosclerotic plaque formation by increasing oxidative stress and decreasing NO production. High phosphate concentrations go hand in hand with increased EMP caused by reduced cadherin levels [32].

Recent studies spotlight the involvement of enzymes called metalloproteinases (MMPs) in the ongoing inflammatory process. These enzymes are responsible for the degradation of extracellular matrix (ECM) components and basement membranes (BMs) of kidneys and blood vessels, among others, in the course of glomerular inflammatory diseases. This process thus facilitates the migration of immune cells to these sites and predisposes to an increase in the rate of development of diseases such as atherosclerosis. MMPs activity rely on the presence of surrounding metal atoms such as zinc, and the levels of these enzymes are significantly increased during the inflammatory process. MMP-2 and MMP-9, which belong to this group of enzymes, are believed to be responsible for disrupting the integrity and tissue architecture of glomerular structures, as they degrade the crucial component of the ECMs in this region, namely, the type IV collagen. In addition, science provides us with an increasing amount of evidence that these enzymes are involved in the pathogenesis of inflammation of the lungs, joints, and smooth muscles, as well as cardiovascular disease, particularly atherosclerosis. It is speculated that the discovery of a substance that inhibits the activity of MMPs could bring vast benefits in the prevention and treatment of diseases that are dependent on these enzymes, and the aforementioned MMP-2 and MMP-9, along with other active products of adipose tissue such as tissue inhibitor of metalloproteinase (TIMPs-1), ox-IDL, high-sensitivity C-reactive protein (hsCRP), or isoprostane-8 and -15, could be considered as therapeutic targets with the potential to contribute to the regression of vascular disease and atherosclerosis at any stage of disease development [34,35].

Once the kidney building cells are damaged, inflammation develops and is steadily increased by migrating immune cells. They produce chemokines such as tumor necrosis factors (TNFs) and monocyte chemoattractant proteins (MCP), which mediate the process of fibrosis through the proliferation of the ECMs. Under homeostatic conditions, tubular epithelial cells and mesangial cells produce low levels of MMP-2 and -9. The result of fibrosis in the kidneys is the activation of multiple pathways that have the effect of increasing the production of the aforementioned MMPs. There are two factors affecting the transition of pro-MMPs to MMPs, namely, plasmin and urokinase plasminogen activator (UPA), the levels of which are increased by interstitial fibrosis. Tissue inhibitor of metalloproteinases (TIMPs) plays a dual opposing role. Four types of this inhibitor can be distinguished, for instance, type 2 activates MMP-2 at low concentrations, whereas when its level is too high, it is responsible for its inhibition. TNF-α and TNF-β, as proinflammatory chemokines, contribute to the process of renal interstitial fibrosis. In addition, they interact with MMP-2 and -9, inducing their expression; however, those chemokines additionally promote pro-MMP activation. Monocyte chemoattractant proteins alongside tumor transforming factors also have a role in fibrosis progression. There are five types of MCPs, although studies have shown that MCP-1 increases the activity and expression of MMP-2 and -9. An interesting phenomenon is that the produced MMP-2 is able to deactivate MCP-3, which then acts as an antagonist for chemokines and inhibits the influx of inflammatory cells. This gives us a new insight into patients with congenital deficiency of MMP-2. In these patients, there will be no inhibition of receptors for chemokines; thus, there will be an increase in the secretion of secretory phospholipase A2(SPLA2), which plays an important role in the development of kidney disease. Another group of factors affecting fibrosis are epidermal growth factor (EGF), fibroblast growth factor (FGF), platelet-derived growth factor (PDGF), transforming growth factor (TGF), and connective tissue growth factor (CTGF). Similar to all of the above, these factors are also related to the MMPs in question. For example, MMP-9 increases TGF-β expression, and MMP-2 has an activating effect on FGF, both of which have a stimulatory effect on fibrosis. Increased MMP-2 activity in the early stages of CKD causes degradation of type IV collagen in the renal basement membrane. This entails an increase in the expression and activity of TGF-β and collectively leads to a process of fibrosis. When the stage of CKD is advanced, a decrease in extracellular matrix deposition occurs due to a reduced activity of MMP-2. One possible explanation for this condition is the dysfunction of oxygen diffusion in the renal tubules, which have been fibrosed. At this point we reach yet another aspect of the disease affecting the fibrosis of the organ. Dead cells cause an influx of neutrophils in an endeavor to digest them. As a consequence of cell phagocytosis, oxidative stress arises, which, depending on the stage of CKD, causes either an increase or decrease in MMP-2 and -9 activity [34,35,36].

Chronic kidney disease is a condition in which the natural regenerative mechanisms do not keep pace with the progressive damage to the vascular endothelium. Therefore, the prevention of urological diseases and prevention of progressive cardiovascular effects are crucial.

#### 4.1.3. Autoimmune Diseases

Autoimmune diseases are caused by a loss of immune tolerance to the body’s own antigens, and the prevalence of these diseases is increasing worldwide. Autoantigens, which induce tissue inflammation, autoreactive T cells, and autoantibodies, play a key role in autoimmunity. Recent studies have highlighted the pathogenic role of Th17 cells and T follicular helper cells (Tfh cells). Proper activation of Th17 and Tfh cells protects the body from infection, but uncontrolled production of these cells can also contribute to the development of autoimmunity [37].

The prevalence of atherosclerosis is higher among patients with autoimmune diseases. Patients with autoimmune diseases such as psoriasis, rheumatoid arthritis (RA), and systemic lupus erythematosus (SLE) tend to have an increased cardiovascular risk and a worse prognosis for cardiovascular events [38]. Furthermore, cholesterol-lowering therapies such as low-fat diets or statins have been shown to be effective in alleviating autoimmune symptoms [39]. Nevertheless, the cellular and molecular mechanisms by which atherogenic factors contribute to the pathogenesis of autoimmune diseases are not well defined. Since atherosclerosis is caused by an imbalance in lipid metabolism, it can be speculated that the hyperlipidemic in vivo environment induced by deregulated lipid metabolism is involved in the pathogenesis of autoimmune diseases associated with hyperlipidemia. Systemic autoimmune diseases and atherosclerosis share common pathogenic pathways. Moreover, autoantibodies (a necessary component of autoimmune disease) can cause damage to the arterial wall and subsequent development and rupture of atherosclerotic plaque. In rheumatic diseases, atherosclerosis may be accelerated by both systemic inflammation and local vasculitis. The presence of an inflammatory autoimmune disease is an indication for early and regular monitoring of the lipid profile and should be included in cardiovascular risk stratification [40]. This issue is so important that the European Alliance of Associations for Rheumatology (EULAR) has developed recommendations for cardiovascular risk reduction in diseases such as systemic lupus erythematosus, rheumatoid arthritis, psoriatic arthritis, vasculitis, systemic scleroderma, myositis, mixed connective tissue disease, Sjögren’s syndrome, and antiphospholipid syndrome [41].

It is well documented that innate immune cells, including macrophages and dendritic cells, detect types of lipids, such as saturated fatty acids and oxidized low-density lipoproteins, and produce proinflammatory cytokines and chemokines [37].

##### Rheumatoid Arthritis

The mechanism leading to rheumatoid arthritis is inherently inflammatory and facilitates the co-occurrence of other immune-inflammatory conditions, including atherosclerosis. The pathophysiological connection between RA and atherosclerosis is rooted in intricate inflammatory pathways that link the two conditions and serve as an elucidation for the increased cardiovascular morbidity in RA patients. TNF-α is a proinflammatory cytokine whose concentration in synovial fluid is significantly elevated in RA patients. TNF-α, together with interleukin-6 (IL-6), promotes the accumulation of oxLDL in the vessel wall, which directly contributes to the formation of lipid-laden macrophages. Another RA-associated cytokine that has proinflammatory properties similar to TNF-α is interleukin-1 (IL-1). Both promote the expression of adhesion molecules on vascular endothelial surfaces, induce cytokine production, and spur the expression of proinflammatory genes, all of which contribute to the development of atherosclerosis [42,43,44,45].

##### Systemic Lupus Erythematosus

In patients with SLE, levels of circulating apoptotic endothelial cells are elevated and are strongly associated with vascular dysfunction and elevated tissue factor levels, suggesting a significant imbalance between vascular repair and endothelial cell damage in systemic lupus erythematosus [46].

Overactivation of the endothelium in SLE patients occurs presumably through increased levels of adhesion molecules on the endothelial surface, as well as through the action of autoantibodies and the formation of oxidatively modified lipids in the inflammatory environment. Platelets, activated by circulating immune complexes, release a host of mediators that cause endothelial dysfunction. IL-1β is believed to be the main factor responsible for excessive endothelial activation and expression of IL-6 and IL-8 cytokines and adhesion molecules. In addition, proinflammatory HDL (piHDL), which is common in SLE patients, increases oxLDL levels and enhances the inflammatory response. The dysregulated immune response in SLE promotes monocyte adhesion to the endothelial monolayer through the mediation of scavenger receptors. This results in the formation of lipid-laden macrophages [47].

Patients with SLE also have elevated levels of circulating autoantibodies, which contributes to acceleration of atherosclerosis development. Although anti-oxLDL and antiphosphorylcholine IgM antibodies typically have a protective effect against atherosclerosis, anti-oxLDL IgG antibodies are atherogenic and cause further progression of SLE. Antiphospholipid antibodies (aPL), namely, lupus anticoagulant, anticardiolipin antibodies, and antibodies against β2-glycoprotein I (anti-β2-GPI), are found in approximately 20–30% of SLE patients, and their presence is related to an increased risk of arterial and venous thrombosis [47,48]. Studies have shown that β2-GPI, in particular, can induce specific reactivity of T cells, and this is highly expressed in the subendothelial regions and in the intima-media complex of human atherosclerotic plaques. The reactive T cells specific for β2-GPI may promote proinflammatory states and the release of pathogenic antibodies. Furthermore, β2-GPI also couples with oxLDL, forming oxLDL/β2-GPI complexes that may enter macrophages through phagocytosis, leading to an increase in foam cell transformation and subsequent atherosclerotic plaque formation. The increased levels of TNF-α and antilipoprotein lipase antibodies seen in SLE may contribute to dyslipidemia by suppressing the activity of lipoprotein lipase, which is needed in order to hydrolyze triglycerides in VLDL, chylomicrons, and other lipoproteins [47].

##### Systemic Sclerosis

In systemic sclerosis (SSc), an overproduction of collagen occurs as a result of increased fibroblast activity and endothelial cell activation, which leads to diffuse microangiopathy. At the same time, microvascular functional and structural changes are the hallmarks of vasculopathy associated with SSc. On the other hand, atherosclerotic macroangiopathy further worsens the prognosis of patients with SSc [49].

It is commonly acknowledged and well documented that endothelial dysfunction plays a role in the pathogenesis of both atherosclerosis and SSc. Increased levels of endothelin, the most puissant vasoconstricting peptide secreted by endothelial cells, are crucially responsible for endothelial dysfunction in both of these diseases. However, studies indicate that endothelin-1 (ET-1) partakes in fibroblast activation as a downstream target of TGF-β, thereby representing one of the rationales for targeting endothelin in SSc patients [50].

The study conducted by Schiopu E et al. showed that patients with SSc had more atherosclerotic plaques (45.6% vs. 19.5%, *p* = 0.01) but similar carotid intima-media thickness (CIMT), as compared to controls. Multiplex analysis revealed significant correlations between serum inflammatory proteins, vasculopathy, and fibrosis with atherosclerosis in SSc. Revealed dependencies included various elements such as IL-2, IL-6, CRP, keratinocyte growth factor, intercellular adhesion molecule-1, endoglin, plasminogen activator inhibitor 1, and insulin-like growth factor binding protein-3 associated with carotid atherosclerotic plaque. Myeloid progenitor inhibitory factor 1, serum amyloid A, thrombomodulin, N-terminal pro-brain natriuretic peptide (BNP), and Clara cell secretory protein 16 kD correlated with carotid intima-media thickness. The median composite score for patients with plaques was 6, while the group without plaques obtained 2 (*p* < 0.0001) [51].

##### Systemic Vasculitis

The most prominent systemic large vessel vasculitis include Takayasu’s arteritis (TAK) and giant cell arteritis (GCA). They are characterized by vascular infiltrates with granulomatous inflammation and the presence of multinucleated giant cells, resulting in tunica media destruction and intimal hypertrophy. Despite the fact that their histopathology largely overlaps, TAK is usually characterized by a lower CD4/CD8 ratio and an increased number of natural killer (NK) cells compared to GCA [52]. Although both are traditionally considered cell-mediated diseases, a role for B cells has recently been implicated. The presence of autoantibodies against endothelial protein C receptor and scavenger receptor class B type 1 has been observed in TAK patients, suggesting a possible role of humoral immunity in this disease [53].

Vasculitis affecting medium-sized arteries includes polyarteritis nodosa (PAN), which is characterized by systemic necrotizing vasculitis in the absence of antineutrophil cytoplasmic antibody (ANCA). It is most often caused by infection (especially hepatitis B, human immunodeficiency virus (HIV), or streptococcus). Small-vessel inflammation includes ANCA-associated vasculitis, which includes three different diseases: granulomatosis with vasculitis (GPA, formerly known as Wegener’s granulomatosis), microscopic polyangiitis (MPA), and eosinophilic granulomatosis with vasculitis (EGPA, formerly called Churg–Strauss syndrome) [52].

Accelerated atherosclerosis in the course of systemic vasculitis occurs from direct vascular inflammation and systemic inflammation, as well as the coexistence of traditional cardiovascular risk factors (such as hypertension, dyslipidemia, renal failure, and reduced physical activity) and the impact of the treatment used. The ongoing inflammation in the vasculature resulting from autoimmunity causes a direct effect of vascular infiltration on the arteries. This leads to increased endothelial leukocyte adhesion molecules, endothelial dysfunction, activated macrophages in the vessel wall and vascular remodeling with luminal stenosis, occlusion, or aneurysm. Systemic inflammation stimulates additional production of proinflammatory cytokines (IL-1b, IL-6, TNF), endothelial leukocyte adhesion molecules, growth factors (VEGF, PDGF), myeloperoxidase, microparticles, autoantibodies, and neutrophil extracellular trap (NETs) [52,54].

##### Sjögren’s Syndrome

Sjögren’s syndrome (SS) is a chronic inflammatory autoimmune disorder of an unknown etiology in which lymphocyte infiltration of the exocrine glands and impairment of their function occurs, as well as inflammatory changes in multiple organs and systems. Novella-Navarro M. et al. demonstrated that primary Sjögren’s syndrome is an independent risk factor for early vascular damage [55].

Patients with SS have been shown to have increased levels of circulating endothelial microparticles and endothelial integrins, but also alarmins such as S100A8/A9. The latter appears to be associated with SS disease activity. Functional impairment of the endothelial layer and increased thickness of the arterial tunica intima also results from the presence of SS-specific antibodies, i.e., anti-SSA/SSB, in the serum. Of significance is also leukopenia, which is found in patients with more active variants of the disorder and with higher risk of cardiovascular events [56].

Research conducted by Balarini GM et al. indicated that a marker of atherosclerosis in SS is calprotectin, which is a complex of S100A8 and S100A9 proteins highly expressed in neutrophil cytoplasm [57]. Increased concentration of S100A8/A9 in SS patients was observed both in glands, where it correlated with the severity of the clinical condition, and in the circulation, where it was associated with increased production of IL-1β, IL-6, TNF-α, IFN-γ, IL-10, IL-17A, and IL-22 [58].

Gravani et al. [59] described the involvement of the Wingless (Wnt) signaling pathway in the development of atherosclerosis. A specific role is played by the soluble inhibitor of the Dickkopf WNT 1 (DKK1) signaling pathway, which is overexpressed on endothelial cells and atherosclerotic plaques, where it enhances the interaction between platelets and the endothelial layer, thereby inducing local inflammation and facilitating atherosclerotic plaque destabilization and its rupture [59,60]. Moreover, plasma DKK-1 levels correlate with hs-CRP and are independent predictors of cardiovascular events [61].

Compounds interconnecting the inflammatory pathogenesis of the SS glandular infiltration and the inflammatory component of the atherosclerotic lesion of the arterial wall also include a complex, consisting of the purinergic P2x7 receptor (P2X7R) and inflammasome NLRP3, which is involved in the pathogenesis of focal inflammation in SS through caspase-1-mediated release of IL-18 [62].

##### Antiphospholipid Syndrome

Cardiovascular events are a major cause of morbidity and mortality in antiphospholipid syndrome (APS). They are a result of thrombotic inflammation and atherosclerosis induced by antiphospholipid antibodies (APLs), which include lupus anticoagulant (LA), anticardiolipin antibodies (ACAs), and anti-β2 glycoprotein-I antibodies (anti-β2GPIs). They engender endothelial dysfunction by inhibiting endothelial NOS and therefore impairing both NO production and its release via apolipoprotein E receptor 2 (apoER2) and protein phosphatase 2A (PP2A) [63]. APLs, in particular ACA and anti-β2GP1, induce aforementioned alterations through activation of the phosphatidylinositol 3-kinase AKT pathway, followed by the rapamycin complex (TORC) [64].

Patients with APS, besides endothelial dysfunction, accelerated proliferation of endothelium, and tunica intima in general, also suffer from platelet activation, release of inflammatory products, and disruption of coagulation and fibrinolysis [65].

Annexin A2 (ANXA2) complex, which facilitates plasmin production on the surface of endothelial cells, is involved in the pathogenesis of atherosclerosis. In APS, underexpression and/or impairment of ANXA2 occurs, which may give rise to thrombosis. In immunocompetent cells, ANXA2 is engaged in membrane repair, vesicle fusion, and organization of the cytoskeleton, thus performing a fundamental role in proinflammatory response and tissue damage [66].

In APS, the complement system is activated either with C5b-initiated formation of a membrane attack complex, which triggers proinflammatory signaling pathways and, through binding C5a with endothelial cells, increased neutrophil adhesion, expression of tissue factors, and release of other procoagulant agents [64,67].

#### 4.1.4. Cancer

Cardiovascular disease and cancer are the two most common causes of death worldwide. Patients who face cancer and oftentimes intensive systemic treatment are subjected to tremendous cardiovascular impact. It is extremely important to know the impact and consequences of cancer on the cardiovascular system at various stages.

Sturgeon et al. [68] performed an analysis of cardiovascular mortality in cancer patients in the US. Sixty-one percent of the cancer patients who died from cardiovascular disease were diagnosed with breast, prostate, or bladder cancer. It was observed that the highest risk of death occurs one year after cancer diagnosis and is negatively correlated with age. This is most likely due to the preexisting cardiovascular burden and the intensification of treatment at younger ages. Compared to the US population, all patients regardless of cancer type have a higher risk of cardiovascular death [68,69]. It has also been shown that patients with endometrial cancer have a significantly higher risk of cardiovascular death in the first year after diagnosis than patients with cancers of other sites. This constitutes, in addition to young patients with intensive treatment, a group of patients in whom early oncologic–cardiovascular collaboration is indicated [68]. It should be noted that the increased cardiovascular risk in this group is not associated with traditional risk factors [69].

Toll-like receptors (TLRs) are found on the surface of many immune cells, but not only there. TLR2, TLR4, and TLR9 receptors are found on the surface of vascular endothelial cells where they participate in the development of its dysfunction and, thus, the formation of atherosclerosis. TLR stimulation triggers a number of signaling pathways ultimately leading to the activation of NF-κB, whose role, among other things, is to regulate the expression of genes such as those for proinflammatory cytokines [70].

In breast, pancreatic, lung, and colon cancers, an increase in TLR activity is observed, which correlates with a worse prognosis. The induced inflammatory stimulation affects the body’s normal cells, which eventually transform into cancer cells. The high-mobility group protein-1 (HMG-1) is produced by activated cells of the immune system and binds to TLR2 and TLR4. When the fusion of these receptors with HMG-1 occurs, it results in the release of cytokines contained in macrophages. Activation of TLR4 itself activates the production of IL-1 through the NOD-like receptor protein 3 (NLRP3), which also enhances the inflammatory process necessary for the formation of atherosclerotic plaques [70].

Prizment et al. proved that breast cancer cells lead to the oxidation of low-density lipoprotein, which results in greater reactivity of LDL against arterial endothelium. Another aspect leading to increased initiation of atherosclerotic plaque is an increase in cell adhesion markers. An increase in the number of relevant ligands on monocytes and their integration with adhesion factors results in increased infiltration of these immune cells under the vascular endothelium. They then undergo a more rapid transformation into foam cells, which demonstrate increased expression of the lectin-type oxidized LDL receptor 1 (LOX-1), resulting in increased uptake of oxidized low-density cholesterol. The presence of monocytes with an M1 phenotype, which neutralize tumor cells, correlates positively with patient prognosis, but also with an accelerated rate of atherosclerotic plaque formation given the proinflammatory effects of M1 monocytes [71].

#### 4.1.5. Obesity

Adipose tissue, as an endocrine organ, is a source of substances that actively affect the functioning of the body. Secreted adipokines and proinflammatory cytokines, such as TNF-α, IL-6, leptin, and resistin, affect the lipid profile, fasting glucose levels, and endothelial function. Hypertrophic adipocytes also release exosomes, which, besides serving auto- and paracrine functions, are involved in the formation of atherosclerotic plaques [72]. Müller showed that exosomes released into the bloodstream are responsible for endothelial damage [73]. Moreover, they regulate lipid deposition and inflammatory responses in macrophages [72]. Abraham et al. reported that visceral adipose tissue (VAT) is much more likely to increase cardiovascular risk when compared to subcutaneous adipose tissue (SAT) [74]. It is also more metabolically active, has a greater capacity to store triglycerides, and has a greater capacity to release more inflammatory cytokines and adipokines than SAT. Exosomes may also be responsible for nonalcoholic fatty liver disease [72].

As mentioned earlier, the formation of atherosclerosis involves M1 macrophages, which are characterized by a more proinflammatory effect, and it is the exosomes that influence macrophage polarization in this direction. Observations also suggest that VAT-derived exosomes also affect the decreased expression of ATP-binding cassette transporters A1 and G1 (ABCA1 and ABCG1), which are responsible for the cholesterol efflux from foam cells to HDL [72].

Bondareva et al. demonstrated that endothelial cells (ECs) from different locations respond differently to obesity. For example, the liver, as a major metabolic center, is adapted to transport inter alia lipids. Fatty acids increase the expression of Fab1p protein responsible for transmembrane transport. In obese individuals, the physiological consequence of increased adipose tissue volume and other tissues is increased angiogenesis. Observations show that this process is impaired in overweight patients. Necrosis of the superficial EC layer occurs, which promotes inflammation, one of the main factors in the formation of atherosclerotic plaques. As obesity progresses, the activation and adhesion of platelets increases. They are supposed to neutralize the emerging necrosis on the EC surface [75].

Although it is known that obesity increases the risk of type 2 diabetes and cardiovascular disease, there is some evidence suggesting that certain disease groups of obese patients may have longer life expectancy or lower mortality rates when compared to their normal-weight peers. This phenomenon has been named as the obesity paradox and is particularly evident in elderly patients with coexisting hypertension, coronary artery disease, or heart failure. One of the many theories attempting to explain this phenomenon is the metabolic reserve hypothesis, which assumes that excess adipose tissue can, in the event of illness or stressful situations, act as a source of additional energy. In contrast, other theories suggest that this phenomenon may be related to the reduced peripheral vascular resistance and lower activity of the renin–angiotensin–aldosterone (RAA) system observed in obese individuals compared to lean individuals. The literature also points out that the mentioned paradox largely affects people suffering from obesity who receive treatment that has a beneficial effect on reducing autonomic system activity (beta-blockers) and modifies neurohormonal responses (angiotensin-converting enzyme inhibitors) in the human body. It should also be kept in mind that individual genetic differences among people with obesity also translate into their ability to compensate for the effects of excessive weight on their health.

Regarding the obesity paradox, it is also worth mentioning that over the past few years a number of interesting papers have been produced indicating possible connections between obesity and cancer. For instance, in a paper written by Wang et al., authors observed that obesity accelerates the aging of the immune system and causes cancer progression directly as a result of metabolic and hormonal dysregulation of the body, as well as through the induction of immunosuppressive pathways [76]. Most notably, their research has shown that obesity leads to T cell dysfunction which results in greater expression of programmed death 1 (PD-1) receptor and increased effectiveness of the blockade between PD-1, and programmed death ligand-1 (PD-L1), which accounts for greater efficacy of anticancer therapy with PD-1/PD-L1 inhibitors and results in the higher patients’ survival rate. On the other hand, Farag et al. pointed out that despite the existence of several more related studies, the immune responses to solid tumors are heterogeneous, due to the different types and even nonidentical subregions of a given tumor [77]. Moreover, due to the heterogeneity of the obesity, its effect on an antitumor immunity is vague and shows discrepancies in cohort studies conducted on patients with cancer of the same type.

Despite many theories, the obesity paradox and its impact on immunological mechanisms still remain poorly understood and mostly unexplained. The controversy stirred up by the topic represents an important field for the development of scientific research aimed at a better understanding of the relationship between health and body weight [78,79].

#### 4.1.6. HIV Infection

Human immunodeficiency virus (HIV) infection is one of the most serious public health threats on a global scale. Since its identification in the 1980s, the virus has become the cause of a global epidemic, affecting the lives of millions of people around the world. HIV attacks cells of the immune system, especially CD4^+^ lymphocytes, weakening the body’s ability to fight infection. Without appropriate treatment, this leads to progressive degradation of the immune system and the onset of acquired immunodeficiency syndrome (AIDS). Nowadays, thanks to widely available antiretroviral therapy (ARV), it is possible to control and reduce the level of viremia in the patient’s blood to undetectable levels, which prevents further damage to the immune system and delays the progression of HIV infection to AIDS [80].

Unfortunately, the pathogenesis of atherosclerosis in the course of HIV infection is still poorly understood at the moment. However, several studies have shown that HIV-infected patients have a higher risk of developing cardiovascular disease. It is speculated that increased translocation of microorganisms from the gut resulting from impaired intestinal barrier, continuous HIV replication, and associated progressive immune deficiency, along with traditional risk factors for atherosclerosis, contribute to immune cell activation and chronic inflammation. Proteins found on the surface of envelope, and encoded by HIV genetic material, stimulate endothelin and IL-6 production and induce inflammation and endothelial dysfunction. Reduced numbers of CD4^+^ lymphocytes are associated with increased intima-media thickness of the carotid artery and arterial stiffness, which translates into a higher risk of cardiovascular events. Studies have also shown that cytomegalovirus (CMV) coinfection and CMV-specific T cell responses correlate with a higher risk of atherosclerosis. Therefore, prevention, early detection of HIV infection, and prompt initiation of ARVs are of utmost importance in the prevention of cardiovascular disease. However, one should remember to choose medications judiciously, as some of them, especially those of older generations, cause an increase in triglyceride and LDL-C levels [80,81,82,83].

#### 4.1.7. COVID-19

In the blood vessels, the angiotensin-converting enzyme 2 (ACE2) is found, which in its transmembrane form is a receptor for the SARS-CoV-2 virus, enabling it to enter the human cell. The virus utilizing the aforementioned enzyme subsequently leads to a reduction in its primary function. ACE2 is an intrinsic inhibitor of the RAA system. This system serves a variety of functions in the organism, while the ACE2 enzyme, converting angiotensin 2 to angiotensin 1, prevents the formation of atherosclerosis, among other things. Deficiency of ACE2 results in an increased angiotensin II blood concentration, and consequently leads to the opposite effects to those found in a homeostatically undisturbed organism. During COVID-19 infection, on the basis of excessive activity of the RAA system, vasoconstriction, as well as the expansion of the inflammatory process and remodeling of the vessel wall occur, which, combined with the emerging hypertension, provide us with a whole range of risk factors for atherosclerosis. On top of this, other abnormalities may arise, such as hypernatremia, hypokalemia, accelerated tissue fibrosis, tissue inflammation, and tissue proliferation [84,85].

The vascular endothelium carries out regulatory activities in the processes of coagulation and fibrinolysis. Endothelial nitric oxide synthase is responsible for regulating vascular tension by producing NO. In addition to NO, prostaglandins also influence vasodilation. SARS-CoV-2 infection is undeniably a generalized inflammation of the body, as a consequence of which NOS function is disrupted through increasing oxidative stress. The nascent viral infection causes dysregulation of the endothelium at multiple levels. Apoptosis of endothelial cells might happen as a result of ROS activity, which results in the recruitment of lymphocytes and platelets, causing prothrombotic and proinflammatory effects, and, additionally, vasoconstriction caused by NO deficiency. The cytokine storm initiated by SARS-CoV-2 leads to the exposure of the deeper layers of the vasculature, and the adhesion factors located in there combine with immune cells and platelets to form clots. The result of the cytokine storm is the formation of disseminated intravascular coagulation (DIC) syndrome, which further stimulates interleukin production that amplifies the cytokine storm therefore forming a vicious circle [84,85,86].

Vascular endothelial walls, on the surface of which atherogenesis has begun, have oxLDL which serves as a molecule with some specificity for TLR4 receptors. Immune cells, expressing TLR4 and TLR2 in response to SARS-CoV-2 virus itself, and fragments of damaged endothelial cells initiate the expression of myeloid differentiation primary response protein-88 (MyD88), a protein responsible for signaling in the inflammatory process. The literature refers to this protein as the interlink between the formation of atherosclerosis and the pathogenesis of COVID-19. The linkage between COVID-19 and atherosclerosis is also indicated by the fact that most of the ACE2 is found in the areas of the endothelium where the highest concentration of cholesterol is. Moreover, macrophages that are already packed with cholesterol activate the NLRP3 inflammasome, which participates in the atherosclerosis development and cytokine storm. The final element that ought to be mentioned is the potential damage caused to the endothelial glycocalyx by the penetrating virus, which disrupts the protective function of the innermost layer of a vessel and uncovers adhesion molecules [85,87].

### 4.2. Nutritional Deficiencies

The complexity of the immunological and nutritional interactions is immense. The efficiency of the immune system is affected by the overall nutritional status and the type of food ingested (foods, nutrients, non-nutritive bioactive compounds). Contrariwise, the immune system regulates metabolism, nutritional necessities, and physiological responses to food [88]. Nowadays, nutrigenomics and immunonutrition (immunomodulatory nutrition) are gaining in popularity, with the aim of improving or maintaining a patient’s nutritional status in preparation for, during, or after treatment [89]. This knowledge is also important in primary and secondary prevention, as modifiable factors, including diet, influence health status by up to 70% [90]. Unfortunately, the current lifestyle of society and the poor food quality mean that we encounter patients with normal or excess body weight, with concomitant deficiencies of non-nutritive bioactive compounds.

It is not only the excess of body weight that is a cardiovascular risk factor. Malnutrition also has a negative impact on the immune system.

#### 4.2.1. Protein–Energy Malnutrition

Protein–energy malnutrition alters both the acquired and innate immune responses. Consequences include decreased bactericidal activity of neutrophils, low levels of complement component 3 (C3) and CH50, decreased NK cytotoxicity, thymic atrophy, peripheral lymphoid tissue atrophy, impaired lymphocyte proliferation to mitogens/antigens, and impaired delayed-type hypersensitivity. Malnutrition decreases proinflammatory cytokines (TNF-α, IL-6, IL-8), which are essential for killing pathogens, whereas anti-inflammatory cytokines (IL-10, IL-33) increase [91,92,93].

Most publications on malnutrition and atherosclerosis focus on patients with chronic renal failure, including those on dialysis [94,95,96]. A study by Okabe et al. confirmed that malnutrition leads to the progression of coronary artery calcification in hemodialysis patients. The study provided important insights into changes in coronary atherosclerotic plaque formation and nonculprit lesion morphology in relation to different nutritional states and standard treatment in patients undergoing maintenance HD. The volume of calcified plaques and calcified nodules was higher in the malnourished state, while the volume of lipid plaques was lower. The analysis revealed that low nutritional status and serum phosphorus levels were independent predictors of progression of calcified plaques [95].

Energy–protein malnutrition promotes the development of atherosclerosis by impairing the function of the immune system, as mentioned above, but also by limiting the supply of building materials for the synthesis of an adequate amount of proteins involved in defense or neutralization of harmful agents. It is difficult to solely consider the impact of energy–protein malnutrition on the development of atherosclerosis. After all, it is often associated with deficiencies of other substances that have a protective effect on the development of atherosclerosis. Restricted food intake is associated with, for instance, reduced intake of foods with antioxidant properties, i.e., fruits and vegetables, or accompanying inflammation [97].

#### 4.2.2. Vitamin Deficiency

##### Vitamin A

Vitamin A is associated with a number of physiological processes. It is actively involved in vision, embryonic development, regulation and differentiation of the immune system, and energy metabolism. The transcriptionally active form of vitamin A, namely, retinoic acid, binds with two types of nuclear receptors: retinoic acid receptors (RARs) and rexinoid receptors (RXRs), and therefore regulates the expression of roughly 700 genes. Retinoic acid is involved in the regulation of biological processes within immune cells, spanning from hematopoiesis to phenotypic changes such as cell polarity [98].

Multiple cross-sectional and case–control studies, using ultrasonographic measurements of the CIMT which is frequently used as a substitutable marker for atherosclerotic cardiovascular disease (ASCVD), have shown that elevated plasma carotenoid levels are associated with a reduced risk of developing ASCVD. The antioxidant properties of both carotenoids and vitamin A could potentially prevent lipoprotein oxidation, one of the early steps in the development of ASCVD [98]. Data analysis of the U.S. National Health and Nutrition Examination Survey (NHANES) indicates a positive association between serum cholesterol concentrations in the HDL fraction and concentrations of α-carotene, β-cryptoxanthin, and lutein/zeaxanthin measured in serum. Moreover, β-carotene and lutein/zeaxanthin levels were negatively related to LDL-C, at the same time that total serum carotenoid values were inversely associated with CRP and total homocysteine, both of which were reported as ASCVD risk factors [98,99]. Retinoic acid regulates the pool of quiescent hematopoietic stem cells (HSCs) and controls immune cell differentiation and proliferation when HSCs are required to respond to specific stimuli, placing retinoic acid signaling at an important position in the hierarchy of immune cell differentiation [98,100]. In ASCVD, macrophages derived from monocytes significantly contribute to atherogenesis progression, as they are the first cells to respond to lipid infiltration of the arterial wall. Increased production of myelocytes causes monocytosis, a condition in which not only more monocytes circulate in a bloodstream, but their infiltration into the arterial wall is also increased, which leads to the progression of ASCVD. Multiple factors facilitate myelopoiesis. Such effects may be observed in hypercholesterolemia, hyperglycemia, and under stress. Retinoic acid is involved in monocyte recruitment to tissues, macrophage differentiation, and the mechanisms that control macrophage seeding in tissues. It also has an impact on T cell differentiation, as it promotes an anti-inflammatory phenotype. In atherosclerosis, Th1 cells promote a formation of atheromatous plaque by promoting monocyte recruitment and polarization of M1-type macrophages with proinflammatory effects. Retinoic acid has an inhibitive effect on Th1 differentiation by reducing the release of IL-12 and its subunit IL-12p40 in macrophages, which promote Th1 differentiation by increasing the expression of the Th1 lineage marker suppressing T-box transcription factor 21 (T-bet). Moreover, retinoic acid inhibits the release of the proinflammatory cytokine IFN-γ by isolated human T cells, which promotes M1 polarization and supports Th1 differentiation in an RAR-dependent manner [98,101]. It is important to note that retinoic acid regulates Th1-related processes in a site-dependent manner. In the intestines, dendritic cells, stimulated by the presence of retinoic acid, secrete IL-12, which increases Th1 cell count and decreases the occurrence of intestinal infections. Stimulation of mature T cells with retinoic acid augments the release of the anti-inflammatory cytokines, predominantly IL-4, IL-5, and IL-13, and facilitates the differentiation of naive T cells into Th2 cells. In addition to promoting the stability of the Th1 cell lineage toward Th17 production and attenuating Th17 differentiation, retinoic acid directly increases the expression of the forkhead box P3 (FoxP3) transcription factor in an RAR-dependent manner. FoxP3 is a determinant of the Treg cell lineage that links vitamin A metabolism and T cell differentiation [98].

##### Vitamin D

Vitamin D is a secosteroid that occurs in the form of cholecalciferol (D3) and ergocalciferol (D2). In order to acquire hormonal properties, it first needs to undergo a cycle of activating transformations. Modifications of its activity happen sequentially: 25-hydroxylation in the liver, where 25-hydroxyvitamin D (25OHD) is formed, followed by 1α-hydroxylation in the kidneys, with the formation of the final form 1,25-dihydroxyvitamin D (1,25(OH)2D). As a steroid hormone in its active form, 1,25(OH)2D multidirectionally marks its participation in cellular metabolic and regulatory processes through intracellular vitamin D receptors (VDR) [102]. Until recently, the vitamin’s main role was attributed to intestinal calcium absorption and bone mineralization, but nowadays, its pleiotropic protective effects are emphasized. The multitude of distinct effects of vitamin D is due to the ubiquity of the VDR in the human body. They can be found in the small intestine, kidneys, osteoblasts, immune cells, pancreas, heart, brain, skin, breast, gonads, prostate, and notably within the cardiovascular system [103].

Vitamin D deficiency is the most common vitamin deficiency in the general population [102], and its effects have been extensively discussed in many publications. Vitamin D indirectly increases antibacterial and antiviral defenses, and affects the function of immune cells of both innate and acquired responses, as well as APCs, which link the two arms of immunity. It enhances the processes of phagocytosis and chemotaxis of neutrophils and monocytes, and inhibits the expression of TLR receptors and proinflammatory cytokines. Furthermore, it induces the production of endogenous antipathogenic peptides in monocytes, neutrophils, and epithelial cells, such as cathelicidin and defensins. Calciferols also inhibit the production of IL-2 and IFN-γ, the two key T cell cytokines. Additionally, they help program tolerance of dendritic cells, and this feature provides vitamin D with a therapeutic application to alleviate symptoms of autoimmune and inflammatory diseases [104]. A prominent representative of multisystem disorders with an immunologic background is systemic sclerosis. Apart from fibrotic processes of the skin and internal organs, it is characterized by endothelial cell dysfunction with vasculopathy. Diaconu AD et al. pointed out the relationship of vitamin D’s immunomodulatory, cardioprotective, and antioxidant properties with the inhibition of the development of systemic scleroderma. These components allow us to hypothesize a plausible positive effect of vitamin D on the course of this disease. Regarding the topic of atherosclerotic lesions, it is important to mention the micro- and macrovascular changes that develop alongside the progression of the disease. The immunosuppressive effect of vitamin D in the course of systemic scleroderma is expressed by inhibition of proinflammatory cytokines (IL-6, IL-17) and stimulation of anti-inflammatory cytokines (IL-4 and IL-10) [103].

Analysis of seasonal blood pressure changes, followed by the discovery of VDR and 1α-hydroxylase in cardiomyocytes, endothelial cells, and VSMCs, indicated the involvement of vitamin D in the activity of the cardiovascular system. The interplay of vitamin D may contribute to the pathophysiology of atherosclerosis by modulating the inflammatory response through decreased expression of inflammatory cytokines such as TNF-α, IL-6, IL-1, and IL-8 in isolated blood monocytes, as well as through an increase in VEGF and decrease in endothelial contractile factors levels [102,103]. Suppression of IL-6 results in reduced synthesis of the acute-phase protein involved in inflammation, namely, CRP. Serum CRP concentrations are associated with atherosclerosis progress and are used as a prognostic indicator of cardiovascular incidents. Vitamin D has been proven to diminish cholesterol deposits in macrophages and LDL uptake. In addition, it modulates the expression of thrombomodulin and tissue factors in monocytes, impacting platelet aggregation and thrombogenicity [102]. In a study carried out by Nakagawa et al., 1,25(OH)2D demonstrated inhibitive effect on the expression of matrix metalloproteinase-2 and -9 (MMP-2 and MMP-9) in cell culture, plausibly counteracting destabilization of atherosclerotic plaque, vascular injury, and thrombosis [105].

##### Vitamin C (L-Ascorbic Acid)

Vitamin C supplementation has been the subject of many reports. However, it should be noted that vitamin C has the same biological activity in both nonoxidized and oxidized forms, i.e., L-ascorbic acid and L-dehydroascorbic acid. It is mainly used in the treatment of deficiency caused by an inadequate diet, the symptom of which is scurvy. Vitamin C is not produced by the human body; therefore, supplementation is necessary. Since both forms of ascorbic acid undergo irreversible oxidation to biologically inactive products in the presence of metal ions Cu^2+^ and Fe^3+^ and oxygen, the problem of supplying a therapeutic dose of vitamin C (200 mg/day) with food arises. In addition, ascorbates that physiologically appear in contact with iron and copper ions show pro-oxidant activity. The combination of aforementioned substances, by stimulating the formation of hydroxyl radical, damages DNA, lipids, and proteins in vitro and in vivo. The physiological concentration of vitamin C, which has an antioxidant effect, is 60–100 mmol/L, while the pro-oxidant effect occurs at an ascorbate concentration of 0.3–20 mmol/L [106]. Simultaneous use of vitamin C with sodium benzoate (a commonly used preservative) causes the latter to be converted into the carcinogen benzene [107].

The protective effect of vitamin C on the prevention of atherosclerosis is due to its action on specific systems. It affects epithelial barrier function by increasing collagen synthesis and stabilization, protecting against ROS-induced damage, and increasing fibroblast proliferation and migration. It further tightens the endothelial barrier by inhibiting protein phosphatase 2A (PP2A) activation and increasing phosphorylated occludin crucial for maintaining tight junctions. On top of that, it affects neutrophils and macrophages by acting as an antioxidant/electron donor, increasing motility/chemotaxis, increasing phagocytosis and ROS production, facilitating apoptosis, and reducing necrosis/NETosis. It improves microcirculatory permeability and protects against pathological vasoconstriction by inhibiting apoptosis of endothelial progenitor cells, inhibiting TNF-α-induced ICAM expression, reducing leukocyte viscosity and aggregation, and preventing eNOS uncoupling and eNO depletion [108,109].

##### B Vitamins

DNA methylation, which modifies a gene without changing its sequence, is an epigenetic mechanism of paramount importance in the pathogenesis of atherosclerosis. Nourishing elements involved in one-carbon metabolism, especially folic acid (vitamin B9) and other B vitamins (B6 and B12), regulate DNA methylation. Deficiencies of these vitamins can increase homocysteine levels, cause endothelial dysfunction, and intensify pathological atherosclerotic processes. Supplementation of nutrients can improve methylation status of DNA, dwindle inflammatory factors, and stop the progression of atherosclerosis. Methylation levels regulate gene expression and disrupt the regulation of pro- and antiatherosclerotic genes. AMP-activated protein kinase (AMPK), consisting of α, β, and γ subunits, takes part in maintaining cellular homeostasis and metabolism. Deletion of AMPKα2 in macrophages may lead to atherosclerosis through an increase in the expression of DNMT1, greater quantitative relation of SAM to SAH (S-adenosyl homocysteine), and amplified methylation of VEGF and matrix metalloproteinase-9 (MMP9) [110].

Homocysteine (Hcy) may trigger endothelial damage with several intracellular mechanisms, including inflammation induction and cell death, disruption of NO production, ROS build-up, prolonged oxidative stress, and cellular hypomethylation. Endothelial damage can be caused by homocysteinylation of proteins through intracellular and extracellular mechanisms. The results of experimental studies conducted on cultured ECs showed that Hcy can induce inflammation through the induction of various inflammatory cytokines, such as interleukin-1β, -6, -8, and -18, and TNF-α, which may result from the accumulation of ROS, activation of inflammasomes, and activation of NF-κB. Hcy may promote EC aging through upregulation of plasminogen activator inhibitor-1 (PAI-1), although telomere truncation and dysfunction may also be a cause of HCy-induced EC aging. EC aging is further accelerated by inflammation and endothelial damage through the secretory phenotype associated with aging (SASP). If Hcy-induced EC damage further exacerbates, the cells may die, resulting in severe endothelial damage. According to a number of studies, Hcy can cause various types of EC death, for instance, apoptosis, pyroptosis, and ferroptosis [111].

The mechanism of disruption of NO synthesis by Hcy Is relatively complex, with an important role played by asymmetric dimethylarginine (ADMA), which is an endogenous inhibitor of NOS. In particular, Hcy post-translationally inhibits the activity of dimethylarginine dimethylaminohydrolase (DDAH), the enzyme that breaks down ADMA. Therefore, Hcy can cause ADMA accumulation and hamper NO synthesis. Hcy can also reduce NOS effectiveness and lessen NO synthesis in eCs through activation of protein kinase C. Reduced NO synthesis leads to endothelial damage by increasing oxidative stress and inflammation [111,112].

Homocysteine is metabolized by transsulfuration to cysteine, then cleaved to sulfate and finally excreted in the urine. Mentioned transformations are regulated by two essential enzymes, CBS and CSE, which may use cysteine as a substrate as well and rely on pyridoxal-5′-phosphate to create hydrogen sulfide. Hydrogen sulfide is an endogenous gasotransmitter that interacts with variously oriented signaling pathways and plays an important role in vascular equilibrium. Downregulation of the CSE/H2S pathway has a major pathological implication in the development of atherosclerosis. It has been reported that H2S regulates many processes that endothelium is involved in, such as angiogenesis, proliferation, and migration [112,113].

Moreover, Hcy has been shown to interfere with lipid metabolism. Hcy may induce buildup of ROS and thus may exacerbate oxidative stress, which can intensify the process of LDL oxidation and have a proatherosclerotic effect [114].

#### 4.2.3. Microelement Deficiency

Microelements are chemical elements found in trace amounts in the body, and their required daily intake is less than 100 mg. They are essential for building purposes (especially in bone tissue), and are included in the composition of body fluids, some enzymes, and high-energy compounds. They also influence the regulation of organ and systemic functions.

##### Zinc

Zinc has some antioxidant properties, affects transcription factors, cell proliferation, and differentiation, DNA and RNA synthesis and repair, activation or inhibition of enzymes, regulation of cell signaling, stabilization of cellular structures, and cell membrane. It is part of more than 300 enzymes. The human body cannot store deposits of zinc [115]. Zinc has a far-reaching role in atherosclerosis development. It can participate in the atherogenic process by interacting with cells directly involved in atherogenesis, such as endothelial cells, VSMC, and immune cells. Additionally, this chemical element also has some considerable, both positive as well as negative, functions in various risk factors associated with atherosclerosis, including lipid metabolism, glucose metabolism, and blood pressure [116].

Zinc regulates nitric oxide (NO) synthesis in the vascular endothelium. Zinc deficiency increases the expression of VCAM-1 and ICAM-1, thus stimulating the inflammation that promotes atherosclerosis. Additionally, it acts on the expression of many proinflammatory cytokines in endothelial cells. Zn deficiency can enable the expression of inflammatory genes in ECs, thereby augmenting the chronic inflammation in the neighborhood of those cells. Recent advances in the microvascular field, described in a systematic review, found that zinc levels are negatively correlated with atherosclerosis and endothelial inflammation development [117]. Zinc deficiency increases NF-κB activity, while zinc supplementation inhibits its activity in ECs [116]. In the development of atherosclerosis, the importance of apoptotic cell death of ECs is highlighted. Therefore, inhibiting EC apoptosis may be an attractive approach to achieve antiatherosclerotic effects. Caspases are crux enzymes in apoptosis pathways, and studies indicate that zinc shows inhibitory effect on caspases [118]. Zinc may also inhibit other regulators of apoptosis besides caspase-3, such as caspase-6, caspase-9, or Ca^2+^- and Mg^2+^-dependent endonuclease [116].

VSMCs are smooth muscle cells that are found in the middle layer of elastic arteries. They control arterial contraction and the production of extracellular matrix (ECM) proteins. VSMCs are a major constituent of atherosclerotic plaque. VSMCs in the plaque have been shown to coexpress biomarkers specific for both VSMCs and macrophages, as well as associated macrophage properties, such as secretion of proinflammatory molecules and induction cell migration. These disclosures suggest a potential divergence in the destiny of VSMCs in atherogenesis, whereas zinc may influence their progression by regulating the function of zinc-finger-containing transcription factors such as KLF4 and ZEB1 [119,120].

Zinc affects macrophage phagocytosis and inflammatory function through a number of regulatory activities and acts as an intracellular secondary messenger in signaling pathways [116]. Excessive cholesterol accumulation in macrophages triggers the inflammasome receptor NOD-like receptor family, pyrin domain containing-3 (NLRP3), a protein that cleaves the interleukin-1 beta precursor to its active form and boosts cytokine expression. Unlike zinc supplementation, TPEN activates NLRP3 inflammasome and increases levels of proinflammatory cytokines, such as IL-1β and IL-6, in macrophages. Inflammatory macrophages hasten the development of atherosclerosis and induce lesion enlargement, causing morphological changes in the atherosclerotic plaque that ultimately lead to plaque rupture and acute luminal thrombosis, which is elicited through proinflammatory cytokines [121,122].

T lymphocytes are preponderantly involved in the progression of the disease, accounting for 10% of all plaque cells, out of which 70% are CD4^+^ T cells, with B lymphocytes found only sporadically in the plaque. Zinc regulates T lymphocytes activity bidirectionally, either through a T cell receptor (TCR-signaling pathway) or through cytokine stimulation [116,123,124].

##### Selenium

One of the essential trace elements, selenium, is required for many metabolic processes, including sustainability of cardiovascular function and protection against improper ROS quantity. Many cardiovascular diseases, including heart failure, ischemic heart disease, myocardial infarction, and atherosclerosis, are linked to selenium deficiency. Se uses selenoenzymes and selenoproteins to oversee redox homeostasis and control free radical damage, Ca^2+^ flow, and thyroid hormone metabolism. These processes can be disrupted by selenium deficiency [125].

Selenoproteins (selenoprotein-T, -S, and -P) regulate ROS quantity and are important elements in antioxidant and redox biology (methionine sulfoxide reductase (MsrB1), thioredoxin reductase (Txnr), and glutathione peroxidase (GPX). Low Se intake results in an impaired stress-induced selenoprotein synthesis, which subsequently leads to an increase in oxidative stress generation and ultimately inflammation, with further implications for cardiovascular system and overall health [126].

Selenoproteins can influence key processes in the development of atherosclerosis, such as oxidative stress created as a result of increased ROS generation, inflammatory reaction (eicosanoid metabolism, immune cell adhesion and migration, and lipid-laden macrophage formation), vascular calcification, apoptosis of vascular cells, and dysfunction of endothelium (normalization of NO levels) [127].

#### 4.2.4. Macroelement Deficiency

Macroelements in the form of minerals serve a number of very important purposes in our body. They are the basic components of the skeletal system structure (e.g., bones and teeth); furthermore, they maintain osmotic fluid balance (water exchange and distribution in the body). In addition, they are one of the constituent elements of soft tissues, are responsible for the conduction of nerve impulses, and are important components of many complex substances, such as vitamins, enzymes, hormones, catalysts, and activators of enzymes.

##### Magnesium

Magnesium plays a key role in controlling vascular smooth muscle tension, endothelial cell function, and myocardial excitability, and is therefore essential in the pathogenesis of sundry cardiovascular conditions, such as hypertension, atherosclerosis, myocardial ischemia, congestive heart failure, and cardiac arrhythmias [128,129].

The study by Cambray et al. was the first to identify the role of magnesium as an effect modifier in the relationship between lipid parameters and carotid intima-media thickness in patients with chronic kidney disease. Thus, high levels of LDL and triglycerides affect CIMT only when Mg levels are low. At the same time, high HDL levels are associated with lower CIMT merely when magnesium levels are high. This finding may explain the paradoxical effect of lipid parameters on atherosclerosis in renal patients, whose Mg levels are often modified by the disease [130].

Research by He X et al. showed that MgCl_2_ attenuates ox-LDL-induced pyroptosis of vascular smooth-muscle-derived foam cells through downregulation of the TLR4/NF-κB signaling pathway. The secretion of IL-1β, IL-18, and LDH was also inhibited by MgCl_2_ [131].

On the other hand, Rahnama Inchehsablagh B et al. showed that magnesium supplementation affects the expression of sirtuin 1 (SIRT1), tumor protein P53, and endothelial nitric oxide synthase genes. The product of SIRT1 gene expression is responsible for apoptosis, differentiation, and cell aging, as well as affecting the regulation of carbohydrate and lipid metabolism. It has cardioprotective and hypotensive significance [132].

Low levels of Mg in the circulation can cause endothelial cell dysfunction. Furthermore, Mg is an important cofactor for many enzymes involved in glucose metabolism. Serum Mg deficiency is associated with metabolic syndrome, which may increase the incidence of atherosclerosis. Lastly, magnesium has been shown to inhibit osteogenic differentiation of vascular smooth muscle cells. Low serum Mg levels may increase vascular calcification [133].

##### Calcium

Both foam cell formation and macrophage migration are calcium-dependent. More importantly, Ca^2+^ stimulates phagocytic cup formation in efferocytic macrophages, which is essential for apoptotic cell clearance, through actin polymerization. Furthermore, Ca^2+^ increases the elicitation of anti-inflammatory cytokines in the aforementioned distinctive macrophages. Sundry Ca^2+^ antagonists have demonstrated calcium involvement at multiple stages of macrophage efferocytosis. On top of this, in vitro and in vivo experiments, as well as clinical studies, have shown that Ca^2+^ antagonists can attenuate atherosclerotic plaque formation by preventing lipid accumulation in macrophages and reducing plaque calcification [134].

Inside the cell, Ca^2+^ is released from the endoplasmic reticulum (the main storage site), the mitochondria, and the Golgi apparatus. The influx of extracellular Ca^2+^ occurs through channels and pumps on the cell membrane. Stimulation of transport between the Golgi apparatus may be responsible for additional protein delivery to secretory vesicles, especially in combination with accelerated translation in the rough endoplasmic reticulum, which may explain the excessive secretion of collagen and elastin by VSMCs of the inner layer of the arterial wall characteristic of atherogenesis. According to this “calcium-atherogenesis” hypothesis, increased cytosolic Ca^2+^ in VSMCs may partially explain the increased secretion of matrix proteins by these cells, leading to an increase in atherosclerotic lesions [135].

Scavenger receptors (SRs) are a family of surface-expressing receptors that are involved in the internalization of extracellular components and directing them to lysosomal compartments. SRs are an essential molecules involved in the identification, absorption, and subsequent utilization of modified lipoproteins, especially ox-LDL, and relevantly, several studies have shown that SR activity in the phagocytic process is dependent on Ca^2+^ influx [136,137].

#### 4.2.5. Omega-3 Fatty Acids

Constituents of omega-3 fatty acids (ω-3 FAs) are eicosapentaenoic acid (EPA) and docosahexaenoic acid (DHA). Both of these acids are involved in many different processes in the human body, including regulation of the immune system, reducing the risk of cardiovascular events and anti-inflammatory effects [138].

In addition to their beneficial effects on lowering triglycerides, ω-3 FAs prevent the development of atherosclerosis by inhibiting inflammation and atherosclerotic plaque rupture [139].

ω-3 FAs reduce triglycerides (TG) in a dose-dependent manner through increased fatty acid oxidation and acyl-CoA:1,2-diacylglycerol acyltransferase (DGAT) impediment, thereby attenuating production of VLDL in hepatocytes and lipogenesis. They specifically stimulate the G-protein-coupled receptor GPR120, promoting differentiation of brown and beige adipocytes, thereby causing thermogenic activation. GPR120 binding also induces adipocytes to release fibroblast growth factor-21 (FGF21). ω-3 FAs are also potent peroxisome proliferator-activated receptor (PPAR) agonists. PPARs constitute a subgroup of three ligand-induced transcription factors and are a member of the nuclear hormone receptor family. Thus far, three different PPAR isoforms have been identified in mammals, namely, PPAR-α, PPAR-β/δ, and PPAR-γ. They are part of a greater superfamily incorporating nuclear hormone receptors. PPARs attach to regulatory elements (PPREs), combine with the retinoid X receptor (RXR), and form heterodimers. This fusion results in an active transcriptional complex that controls a multitude of genes related to lipid metabolism. The aforementioned complex controls multifarious aspects of adipogenesis, inflammation, and metabolic equilibrium. PPAR-α activation, which leads to hepatic expression of apolipoprotein A1 and apolipoprotein A2, prompts the decrease in levels of VLDL and TG, while increasing the amount of circulating HDL. Unlike glitazones (PPAR-γ agonists), which induce the expression of lipoprotein lipase in adipose tissue, fibrates (PPAR-α agonists) reduce the amount of TG in the circulation [140,141].

EPA and DHA exert anti-inflammatory effects. They inhibit leukocyte chemotaxis and adhesion molecule expression, control signal transduction by affecting both surface and intracellular receptors, and influence the reactivity of B and T lymphocytes and alter the fatty acid composition of cell membranes [142,143].

However, recent findings show benefits from an EPA medicine known as ethyl icosapent (IPE), but not from more traditional mixed ω-3 FA preparations or other TG-lowering agents. These revelations have sparked curiosity in the differential outcomes of various ω-3 FA preparations, particularly IPE, with respect to reducing the risk of atherosclerotic thrombosis. Compared to other ω-3 FAs, in particular DHA, which is concentrated in neuronal membranes, EPA has different metabolic products, platelet integration, membrane interactions, lipid antioxidant activity, and tissue (i.e., arterial) distribution, according to studies conducted both in vivo and in vitro. EPA maintains the distribution of cholesterol in membranes and perpetuates normal phospholipid packing constraints. Furthermore, EPA contends with arachidonic acid for cyclooxygenase (COX), and improves endothelial function when combined with high-intensity statins compared to DHA. These original biological activities of EPA, together with its intricate bioactive metabolites, may be conducive to new insights into atherothrombotic disease mechanisms and therapeutic interventions [140].

#### 4.2.6. Coenzyme Q10

Coenzyme Q10 (CoQ10), also known as ubiquinone, ubiquinol, 1,4-benzoquinone, ubidecarenone, or, erroneously, vitamin Q10, is a naturally occurring substance in the human body with many roles. CoQ is primarily needed in mitochondria as a transporter of electrons and protons in the mitochondrial respiratory chain (MRC). The MRC, through oxidative phosphorylation, provides cells with the ability to synthesize ATP, which is essential for cell function. CoQ has multiple extramitochondrial effects, including activation of AMP-activated protein kinase (AMPK), regulation of the inflammasome, modulation of mitophagy, membrane peroxidation preclusion, and regulation of the physicochemical properties of cell membranes, due to its lipid-soluble antioxidant potential. In addition, CoQ has been proven to exert epigenetic regulation in genes involved in cell signaling, intermediary metabolism, intracellular transport, transcriptional control, disease mutations, protein phosphorylation, and embryonic development. Furthermore, CoQ may improve endothelial dysfunction and potentially increase cardiac ATP production and cardiac output by exerting a positive inotropic effect on the myocardium. It may also have some antihypertensive effects [144].

CoQ deficiency and mitochondrial dysfunction may be partially involved in the cellular pathophysiology of early atherosclerosis, allowing increased free radical production and activation of inflammasomes in the vascular endothelium [145].

According to the study by Chokchaiwong S and Cordero M.D., the supplementation of CoQ depletes a variety of inflammatory parameters, including NLRP3 activation, mainly by restoring or even strengthening mitochondrial function [146,147]. Atherosclerosis-induced arterial damage is generally ongoing, with an increase in foam cell amount and cytokine production that allows even greater macrophage recruitment in the lesions, which further augment lipid accumulation. According to multiple studies, CoQ lowers foam cell formation rate, which corresponds to reduced lipid and macrophage accumulation [148,149]. Additionally, a study concentrated on the role of macrophages in atherosclerosis disclosed novel methods, using reverse cholesterol transport (RCT), to remove excess cholesterol from peripheral cells and lesions. Plenty of lipid and cholesterol carriers mediate the described pathway. The prominent intermediaries feature cholesterol efflux proteins ABCA1 and ABCG1. CoQ appears to be promoting macrophage RCT via a specific interaction between microRNA and ABCG1. Thus, it contributes to the prevention of atherosclerosis [150].

Coenzyme Q10, a potent activator of AMPK, serves a significant function in atherosclerosis development, as it regulates lipid and carbohydrate metabolisms, but also VSMCs, endothelial cells, and immune cells activity. Hence, atherogenesis is associated with dysregulation of autophagy and reduced AMPK activity through increased production of ROS and inflammatory cytokines in the endothelium. AMPK and autophagy, in general, promote cholesterol metabolism and mitigate atherosclerosis by regulating the expression of cholesterol transporting proteins. Collectively, the evidence suggests that macrophage autophagy takes part in inflammation inhibition, apoptosis, efferocytosis promotion, and cholesterol clearance. In addition, AMPK activation promotes anti-inflammatory effects, such as an increase in IL-10 concentration in endothelium and macrophages, while suppressing proinflammatory cytokine release [151,152,153].

### 4.3. Medicaments

The use of certain medications is associated with an increase in cardiovascular risk. Such adverse effects may be observed with glucocorticosteroids, or anticancer drugs.

#### 4.3.1. Immunosuppressive Medications

##### Glucocorticosteroids

Glucocorticosteroids are steroid hormones that are instrumental in regulating many physiological processes, including cardiovascular homeostasis. Glucocorticoids are synthesized from cholesterol in the cortical layer of the adrenal glands, particularly in the zona fasciculata, and are released in response to stimulation by adrenocorticotropic hormone (ACTH), which is secreted from the anterior lobe of the pituitary gland. This release is regulated by feedback from the hypothalamic–pituitary–adrenal (HPA) axis. In humans, the main glucocorticoid is cortisol, although corticosterone is also produced. Chronic overactivation of glucocorticoid receptors causes obesity, insulin resistance, glucose intolerance, dyslipidemia, and hypertension. Subtle abnormalities in the hypothalamic–pituitary–adrenal axis and/or tissue sensitivity to glucocorticosteroids are also associated with these risk factors for cardiovascular disease in patients with metabolic syndrome. In addition, glucocorticoids exert direct cardiovascular effects via glucocorticoid and mineralocorticoid receptors and are modified by local glucocorticoid metabolism by 11-beta-hydroxysteroid dehydrogenase enzymes. These effects affect vascular function, atherogenesis and vascular remodeling after intravascular injury or ischemia [154,155]. Clinical and preclinical studies have demonstrated both a protective and proatherosclerotic response to glucocorticoids, the effects of which depend on their multifactorial actions. The activity of glucocorticoids on the vascular wall depends on the degree of exposure (both dose and exposure time), the expression of the corresponding receptor, and the bioavailability of the steroid itself and its access to intracellular receptors [156].

Atherosclerotic effects are induced in the endothelium, VSMCs, and macrophages. In the terms of the former, there is a decrease in endothelium-derived relaxing factor (EDRF) generation, an increase in reactive oxygen species production, and a decrease in endothelial nitric oxide synthase activity. In VSMCs, there is an increase in the production of vasoconstrictors such as endothelin-1 and angiotensin II, an increase in angiotensin-1 receptor expression, and an increase in cholesteryl ester accumulation. In macrophages, there is also an increase in cholesteryl ester accumulation and an increased lipotoxicity [156].

The antiatherosclerotic process can also be induced in the endothelium and in macrophages. Regarding the endothelium, a nongenomic increase in eNOS production, a decrease in the expression of adhesion molecules, and the expression of proinflammatory cytokines is observed. In contrast, macrophages show decreased accumulation and expression of proinflammatory cytokines as well as decreased uptake of oxLDL and cholesterol efflux [156].

##### Methotrexate

Methotrexate (MTX), as an antimetabolite and folic acid antagonist, inhibits the activity of dihydrofolate reductase, which catalyzes the conversion of dihydrofolate to tetrahydrofolate. Indications for its use include mainly autoimmune disorders and cancer. MTX acts by inducing apoptosis of activated CD4^+^ T cells and affects the inhibition of Il-6 and -10 production.

Esparza et al. conducted a study on mice exposed to a high-fat diet. Regardless of the MTX dose administered (10, 30, or 50 mg/kg/week), all study groups demonstrated differences in aortic thickness compared to the control group. A significant difference was also observed between the groups treated with the lowest dose (10 mg/kg/week) and the higher dose (30 or 50 mg/kg/week) [157].

Considering the ratio of the vessel lumen to the entire cross-section, a significantly noticeable difference was found in the control group mice, where the ratio was reduced, when compared to the experimental groups, regardless of the dosage [157].

Liu et al. also reported a reduction in the intensity of atherogenesis caused by disturbed blood flow with MTX. MTX reduced the influx of monocytes and the development of inflammation that developed in response to disrupted flow [158].

The YAP protein is a constituent of the pathways responsible for cell proliferation and differentiation. Blood vessels produce large amounts of the YAP protein in response to mechanical stimuli of turbulent blood flow, and this very protein affects the expression of adhesion molecules. Consequently, adhesion of immune cells forming atherosclerotic plaque and thickening of the endothelium occur. Disturbed blood flow leads to decreased phosphorylation and increased activation of the abovementioned protein, resulting in an increased activity of the atherogenesis process. The application of MTX results in reduced YAP expression and decreases platelet development. Statins, which are commonly used in lipid disorders, also affect the YAP protein by reducing its activity and therefore delaying the formation of atherosclerosis [158].

##### Colchicine

Colchicine, used in gout, has anti-inflammatory and antitubulin properties that inhibit neutrophils and has an effect on the NLRP3 inflammasome [159]. Nidorf et al. demonstrated that colchicine reduces inflammation in the form of C-reactive protein independently of aspirin and statins [160]. Subsequently, the effect of colchicine in preventing recurrent acute coronary syndrome and ischemic stroke with satisfactory outcomes was investigated [159].

Mylonas et al. investigated the effects of a hyperlipidemic diet on animal models supplemented with colchicine, monitoring the development of atherogenesis. The researchers reported that in all study groups, colchicine combined with fibrate, and colchicine combined with N-acetylcysteine (NAC), atherosclerotic lesions developed in the thoracic and abdominal aorta. However, the combination of colchicine with NAC resulted in the greatest protection against atherogenesis in each aortic segment [161].

The α-Klotho gene, which belongs to genes that protect against atherosclerosis, had increased expression in both study groups. The Klotho protein is responsible for endothelial integrity, and stops chemokine-induced expression of adhesion molecules, which reduces adhesion of immune cells and platelets. This protein protects endothelial cells and muscle cells from oxidative stress and indirectly increases the amount of glutathione in the body. The literature also reports the possibility of perceiving the Klotho protein as a prognostic factor for early atherosclerosis [161].

The combination of colchicine with NAC, as opposed to the colchicine with fibrate, resulted in a restoration of the HOXA5 gene baseline values, which is also an antiatherogenic gene [161].

##### Other Immunosuppressants

There is a plethora of immunosuppressive drugs available. It is beyond the scope of this paper to discuss all of them. In order to cover patients with the best possible care that takes into account cardiovascular risk, it is necessary to include their usage.

Hydroxychloroquine, an antimalarial drug, in its spectrum of action also exhibits anti-inflammatory properties, and hence may weaken immune cells. Inhibition of the innate immune response in the form of suppression of Toll-like receptor-9 (TLR-9) stimulation also has the potential to weaken the immunological atherogenic factor [159].

The reduction of inflammation is undeniably one of the factors limiting atherogenesis. The anti-IL-1β monoclonal antibody, canakinumab, significantly reduces the levels of C-reactive protein and IL-6. However, the study found no impact of this antibody on lipid values or platelet activity [159].

#### 4.3.2. Cancer Chemotherapy

As the incidence of cancer continues to rise, the cardiovascular complications associated with cancer treatment are becoming a growing problem.

##### Angiogenesis Inhibitors

Since the link between angiogenesis and cancer cell proliferation has been established, therapies aimed at inhibiting angiogenesis have become an integral part of anticancer treatment. Angiogenic factors, which mainly include vascular endothelial growth factors and VEGF receptors, have become targets. Vascular endothelium is the primary target location for angiogenic factors. Angiogenic factors induce vasodilation of blood vessels mediated by the endothelium in order to sustain circulation and stimulate angiogenesis. Mobilization of circulating endothelial progenitor cells from the bone marrow is induced in order to stimulate angiogenesis. To maintain normal blood flow, VEGF promotes the production of NO and prostacyclin (PGI2) in vascular endothelium, which helps in wound healing and in treatment of vascular endothelial damage in healthy adults. VEGF secretion is induced by hypoxia, associated with tissue ischemia in diseases such as ischemic heart disease, resulting in compensatory angiogenesis. In the tumor microenvironment, VEGF produced by tumor cells induces angiogenesis and proliferation necessary for tumor development and spread. Furthermore, VEGF plays an important role in the proliferation and metastasis of tumor tissue. A prime example of a drug with this mechanism of action is bevacizumab, a human monoclonal antibody against VEGF [162]. Angiogenesis inhibitor (AI) effects include increased arterial thrombotic activity, hypertension, increased cardiac fibrosis, and decreased capillary density [163,164]. Ais are thought to act primarily on microvessels 150–200 µm in diameter. Treatment with inhibitors of angiogenesis causes vasoconstriction coupled with a decrease in vascular dilators such as NO and PGI2, resulting in even further vasoconstriction. Moreover, VSMC proliferation, platelet aggregation, thrombosis, and leukocyte adsorption occur in the endothelium, which stimulates endothelial dysfunction and atherosclerotic plaque formation. In contrast, hypoxia and dysfunction of endothelium encourage the endothelin-1 production, resulting in vasoconstriction. Extended usage of angiogenesis inhibitors causes microangiopathy, i.e., anatomic thinning, by promoting a decrease in the peripheral arterial bed and capillary thinning linked to microthrombus development. As a result, elevated blood pressure and thromboembolic disease are induced, culminating in drug-induced atherosclerosis [162].

##### Alkylating Drugs

Alkylating drugs are characterized by a mechanism of action that involves the transfer of alkyl groups to DNA, RNA, and proteins. This phenomenon is referred to as alkylation, hence their name. The alkylation impairs the basic life processes of the cancer cell, eventually leading to its death. Alkylating drugs work regardless of the cell cycle phase [165]. As for cisplatin, induction of endothelial damage, cell apoptosis, stimulation of thromboxane production, platelet activation, and aggregation are highlighted as the main mechanisms of action on the cardiovascular system [166].

##### Antimetabolites

Antimetabolites are modified molecules that inhibit the metabolism of purine or pyrimidine bases from which DNA and RNA are built. Antimetabolites are purely synthetic, phase-specific drugs that are active primarily in the S phase of the cell cycle. Incorporating them into the DNA structure in place of the correct molecules of purine or pyrimidine bases (this occurs by virtue of similarity in chemical structure) or inhibiting enzymes essential for the metabolism of these bases blocks further synthesis of DNA or RNA and thus leads to cell death [167].

An example of a drug representing this group is 5-fluorouracil. Direct damage to the vascular endothelium, followed by thrombosis, characterized by the release of vasoactive substances and vasoconstriction, as well as an alteration in antioxidant defense capacity, are cited as possible causes of the vascular changes caused by this drug [168,169].

##### Drugs of Natural Origin

Drugs of natural origin include anticancer antibiotics, the so-called anthracyclines, such as doxorubicin (DOX) and anthracendiones, e.g., mitoxantrone (MTX), and glycopeptide antibiotics, such as bleomycin. Their action effect is a combination of several mechanisms. These medicaments destroy the DNA structure, generate free radicals, and can directly damage the membrane of the cancer cell. They are cell-cycle-specific substances, with the exception of bleomycin, which acts mainly on the G1 phase [170].

Doxorubicin-induced toxicity is accompanied by an enormous increase in the levels of reactive oxygen species. Excessive Dox-induced oxidative stress leads to the modification of various cellular molecules, including phospholipids. Oxidized phospholipids (Ox-PLs) are involved in the development and progression of several pathologies, including atherosclerosis, thrombosis, and tissue inflammation. Moreover, Ox-PLs and excess iron have been linked to ferroptosis, a form of regulated cell death. Proper neutralization of Ox-PLs increases resistance to ischemia-reperfusion injury, which is related to the preservation of mitochondrial membrane potential. MTX, just as DOX, interacts with iron to generate ROS, targeting topoisomerase 2 and damaging mitochondria. Despite similarities in the way they exert their toxic effects, DOX and MTX appear to differ in one key aspect. DOX is a more redox-disruptive drug, whereas MTX causes an energy imbalance [170,171].

Bleomycin, on the other hand, increases the expression of intercellular adhesion molecule-1 (ICAM-1; CD54), vascular cell adhesion molecule-1 (VCAM-1; CD106), and selectin E (CD62E), as well as the expression of monocyte chemoattractant protein (MCP-1) and interleukin (IL-8) released by endothelial cells. The increase in protein expression is accompanied by increased messenger RNA (mRNA) transcription [172].

#### 4.3.3. Cancer Immunotherapy

The introduction of treatments with immune checkpoint inhibitors (ICIs), which target the CTLA-4 (Cytotoxic T-Lymphocyte Associated Protein 4) and PD-1/PD-L1 (Programmed Cell Death-1/Programmed Death-Ligand 1) pathways, among others, has been a breakthrough in the fight against cancer. Unfortunately, they do not remain without effects on the cardiovascular system, including the development of atherosclerosis [4,173].

Ipilimumab is a fully human anti-CTLA-4 monoclonal antibody used in the treatment of melanoma and non-small-cell lung cancer, inter alia. However, it increases the size of atherosclerotic lesions, causes progression to an advanced atherosclerotic phenotype, and reduces collagen content. Anti-PD-1 monoclonal antibodies (nivolumab and pembrolizumab) and anti-PD-L1 antibodies (atezolizumab and durvalumab) have been found to be effective in the treatment of melanoma, non-small-cell lung cancer, renal cell carcinoma, classical Hodgkin’s lymphoma, squamous cell carcinoma of the head and neck, and urothelial carcinoma. Their proatherogenic effects are manifested by increasing the size of atherosclerotic lesions and TNF-α production, enhancing the activation of lesion-associated helper T cells, cytotoxic T cells, and macrophages [174,175,176].

#### 4.3.4. Radiotherapy

Radiation therapy directly affects cells involved in the pathogenesis of atherosclerosis. It causes endothelial damage with typical symptoms such as increased permeability, expression of interleukins (e.g., IL-6 and IL-8), intercellular adhesion molecules (e.g., ICAM-1), and fibroblast growth factor. Infiltration with monocytes, lymphocytes, and macrophages and activation of lysosomes also occur. The neutrophil response is stimulated with secondary cytokine release. Fibrin deposition occurs due to impaired fibrinolysis, inhibition of transforming growth factor-β, and thrombomodulin. Histologic examination reveals two major complications of arterial irradiation: disruption of the intima and narrowing or obstruction of the arterial lumen. Dyslipidemia, smoking, diabetes mellitus, and hypertension, as well as combined anticancer therapy, may accelerate the process of atherosclerosis formation or aggravate already existing lesions [177].

### 4.4. Smoking

Smoking electronic cigarettes increases the number of nonclassical monocytes, while the number of classical and intermediate monocytes remains constant. Under the influence of e-cigarettes, an increase in the expression of TLR9 is observed, which is responsible for the transformation of monocytes and macrophages into foam cells. Administration of a TLR9 antagonist causes a decrease in fluorescence intensity in all types of monocytes, even more so in classical monocytes, where the primary signal is greatest. An analogous situation applies to the expression of the adhesion molecule VCAM-1 and proinflammatory cytokines. C-C motif chemokine receptors 2 (CCR2s) located on the surface of monocytes under the influence of proinflammatory cytokines affect their activation in atherogenesis. However, the TLR9 antagonist does not affect the adhesion molecule CD62L, which is found on the molecules of T lymphocytes and is responsible for their contact with the endothelium. The use of the aforementioned antagonist also led to a decrease in the deposition of lipids and macrophages in the plaque [178].

Ganapathy et al. proved that the aerosol in e-cigarettes leads to a deterioration of antioxidant defenses in cells [179]. As a result, mitochondrial DNA (mtDNA), which is even more susceptible to damage, is detected in addition to DNA fragments. Fragments of oxidized mtDNA stimulate macrophages to activate TLR9, which promotes atherogenesis, and the process starts all over again [178].

Nicotine is responsible for regulating communication between cells involved in atherosclerotic plaque formation. Nicotine increases the secretion of exosomes derived from macrophages located in the forming atherosclerotic plaque. Exosomes contain large amounts of microRNA (miRNA), which, when secreted into VSMCs, cause their increased proliferation and subsequent migration [180].

## 5. Summary

Atherosclerosis and its complications are a significant global issue, not only due to mortality, reduced life expectancy, and reduced quality of life, but also because of the costs of treatment, rehabilitation, and absence from work.

The article not only elucidates the molecular linkage between the immune system and atherosclerosis, but also provides a clinical guideline on what should be taken into account when assessing cardiovascular risk in patients. When treating patients, we should focus not only on well-known cardiovascular risk factors, such as age, gender, non-HDL concentration, body weight, and smoking, but also include noncardiac chronic diseases (especially autoimmune diseases, cancer, diabetes, and chronic kidney disease), current treatments, and nutritional deficiencies, including foods, nutrients, and non-nutritive bioactive compounds.

Only a holistic approach to atherosclerosis, taking into account modifiable and nonmodifiable risk factors, offers the best chance of reducing cardiovascular risk. Knowledge of the immune system, in particular its mechanisms of action and role in the development of cardiovascular disease, can provide benefits in the form of targeted therapy, delaying the progression and severity of the disease, and, most importantly, prolonging the life expectancy of patients while maintaining a good quality of life.

## Data Availability

No new data were created or analyzed in this study. Data sharing is not applicable to this article.
